# The RhoGAP SPIN6 Associates with SPL11 and OsRac1 and Negatively Regulates Programmed Cell Death and Innate Immunity in Rice

**DOI:** 10.1371/journal.ppat.1004629

**Published:** 2015-02-06

**Authors:** Jinling Liu, Chan Ho Park, Feng He, Minoru Nagano, Mo Wang, Maria Bellizzi, Kai Zhang, Xiaoshan Zeng, Wende Liu, Yuese Ning, Yoji Kawano, Guo-Liang Wang

**Affiliations:** 1 State Key Laboratory for Biology of Plant Diseases and Insect Pests, Institute of Plant Protection, Chinese Academy of Agricultural Sciences, Beijing, China; 2 Hunan Provincial Key Laboratory of Crop Germplasm Innovation and Utilization and College of Agronomy, Hunan Agricultural University, Changsha, Hunan, China; 3 Department of Plant Pathology, Ohio State University, Columbus, Ohio, United States of America; 4 Laboratory of Plant Molecular Genetics, Nara Institute of Science and Technology, Ikoma, Nara, Japan; 5 Department of Science and Technology, Saitama University, Sakura-ku, Saitama, Japan; 6 Signal Transduction and Immunity Group, Shanghai Center for Plant Stress Biology, Shanghai, China; Wageningen University, NETHERLANDS

## Abstract

The ubiquitin proteasome system in plants plays important roles in plant-microbe interactions and in immune responses to pathogens. We previously demonstrated that the rice U-box E3 ligase SPL11 and its *Arabidopsis* ortholog PUB13 negatively regulate programmed cell death (PCD) and defense response. However, the components involved in the SPL11/PUB13-mediated PCD and immune signaling pathway remain unknown. In this study, we report that SPL11-interacting Protein 6 (SPIN6) is a Rho GTPase-activating protein (RhoGAP) that interacts with SPL11 *in vitro* and *in vivo*. SPL11 ubiquitinates SPIN6 *in vitro* and degrades SPIN6 *in vivo* via the 26S proteasome-dependent pathway. Both RNAi silencing in transgenic rice and knockout of *Spin6* in a T-DNA insertion mutant lead to PCD and increased resistance to the rice blast pathogen *Magnaporthe oryzae* and the bacterial blight pathogen *Xanthomonas oryzae* pv. *oryzae*. The levels of reactive oxygen species and defense-related gene expression are significantly elevated in both the *Spin6* RNAi and mutant plants. Strikingly, SPIN6 interacts with the small GTPase OsRac1, catalyze the GTP-bound OsRac1 into the GDP-bound state *in vitro* and has GAP activity towards OsRac1 in rice cells. Together, our results demonstrate that the RhoGAP SPIN6 acts as a linkage between a U-box E3 ligase-mediated ubiquitination pathway and a small GTPase-associated defensome system for plant immunity.

## Author Summary

Rice diseases are the major threat for stable rice production and food security worldwide. Deep understanding of the disease resistance pathway in rice is essential for effective control of the diseases. Although rice contains many E3 ubiquitin ligases, the function of their substrates in immune responses is still not fully understood. We previously characterized U-box E3 ligase SPL11 in rice that is involved in the regulation of cell death, immune responses, and flowering. However, how SPL11 interacts with its substrates to control cell death and immunity is not clear. In this study, we found that the SPL11 interacts with SPIN6, ubiquitinates, and degrades the protein via the 26S proteasome pathway. Both the *Spin6* RNAi and mutant plants show enhanced resistance to rice pathogens and activate defense gene expression and ROS generation. Importantly, we found that SPIN6 is the RhoGAP of the small GTPase OsRac1, which is a key component in rice immunity. Our study provides further insights into the relationship between SPIN6 and its interacting proteins SPL11 and OsRac1, and its function in the control of cell death and immunity in rice.

## Introduction

To resist pathogen invasion, plants have evolved two layers of innate immunity: pathogen-associated molecular pattern (PAMP)-triggered immunity (PTI) and effector-triggered immunity (ETI) [1]. The first layer is PTI, which employs pattern-recognition receptors (PRRs) to perceive a broad range of PAMPs in order to activate basal defense signaling. The second layer, ETI, involves a rapid and robust defense response triggered by the direct or indirect interaction between a pathogen avirulence (Avr) protein and its cognate host resistance (R) protein. A major hallmark of ETI is a strong hypersensitive response (HR), which represents a form of programmed cell death (PCD) and which activates a set of innate immunity signaling pathways that result in ion fluxes [2], generation of reactive oxygen species (ROS) [3], and release of nitric oxide (NO) [4].

The ubiquitin proteasome system (UPS) is involved in selective degradation of proteins in the cells of eukaryotic organisms. It consists of three main kinds of enzymes: ubiquitin-activating enzymes (E1), ubiquitin-conjugating enzymes (E2), and ubiquitin ligases (E3). In plants, many ubiquitin E3 ligases have been implicated in growth, development, and responses to abiotic and biotic stresses [5,6]. The U-box E3 ligase SPL11 is a negative regulator of PCD and defense response in rice [7]. The *spl11* mutant confers broad-spectrum resistance to rice pathogens and has elevated defense gene expression and ROS levels [8,9]. Recently, PUB13 (plant U-box protein 13), one of the SPL11 orthologs in *Arabidopsis*, was also found to negatively regulate PCD and resistance to biotrophic pathogens [10,11], indicating that SPL11-like proteins have conserved functions in both monocot and dicot plants. Interestingly, PUB13 is required for the polyubiquitination and degradation of the PRR FLS2 in the presence of the PAMP effector flg22. The polyubiquitination of FLS2 by PUB12/13 depends on the PUB12/13 phosphorylation mediated by the receptor-like kinase (RLK) BAK1, a component involved in the brassinosteroid receptor BRI1 and in multiple PRR-mediated signaling pathways [12]. These findings suggest that the SPL11- and PUB12/13-mediated protein degradation pathway has a significant role in PTI signaling in plants. However, the components that interact with SPL11 or PUB13 in the control of PCD and defense response remain unknown.

By enhancing active, GTP-bound small GTPases into the inactive GDP state, the small GTPase-activating proteins (GAPs) function as an important regulator in small GTPase-mediated cellular signaling [13]. Like other organisms, plants contain a large number of GAPs [14], most of which are involved in cell morphogenesis and polarization. For example, the rice genome contains 85 GAP genes. Among them, 23 belong to the RhoGAP family [14]. Until now, only a few GAPs have been found to be involved in plant defense signaling. RanGAP2 in *Nicotiana benthamiana*, for example, by directly associating with the nucleotide-binding-leucine-rich repeat (NB-LRR) R protein Rx, regulates the partitioning of Rx in both the cytoplasm and nucleus, and thereby enhances resistance against *Potato virus X* (*PVX*) [15,16]. In barley, microtubule-associated ROP GAP MAGAP1 interacts with a small GTPase RACB and inhibits *Blumeria graminis* penetration by influencing the polar organization of cortical microtubules [17]. OsGAP1 is the only GAP in rice that is known to interact with the small GTPase YchF as a consequence of this interaction, OsGAP1 contributes to resistance to bacterial pathogens when it is constitutively expressed [18,19].

In rice, the small GTPase OsRac1 is a critical defense component because it integrates multiple ETI and PTI signaling pathways [20]. OsRac1 at the plasma membrane (PM) interacts directly with the rice blast NB-LRR R protein Pit and contributes to Pit-mediated ROS production and HR. Furthermore, the active form of Pit activates OsRac1 at the PM [21]. Interestingly, OsRac1 is also activated at the PM by its indirect association with the chitin-receptor complex formed by the chitin-binding protein OsCEBiP and the RLK OsCERK1 [22,23]. A recent report showed that OsRacGEF1, which is a guanine nucleotide exchange factor (GEF) responsible for the activation of small GTPase by promoting GTP binding, activates OsRac1 at the PM by stimulating the formation of active, GTP-bound OsRac1 [23]. In addition, OsRacGEF1 is phosphorylated by OsCERK1, and the activation of OsRac1 is triggered by the association of OsCEBiP/OsCERK1 and OsRacGEF1. However, the components responsible for OsRac1 inactivation remain to be identified.

In this study, we report that the RhoGAP SPIN6 protein interacts with SPL11 and is ubiquitinated *in vitro* and degraded *in vivo* by SPL11 via the 26S proteasome-mediated pathway. Silencing of *Spin6* in transgenic rice causes PCD and enhanced resistance to the blast pathogen *Magnaporthe oryzae* and to the bacterial blight pathogen *Xanthomonas oryzae* pv. *oryzae* (*Xoo*), suggesting that *Spin6* plays a negative role in the regulation of PCD and immunity in rice. Strikingly, we found that SPIN6 interacts with OsRac1 and catalyze the active form of OsRac1 into an inactive form in rice cells. Together, these results show that SPIN6 is a key molecule that transduces defense signals from the E3 ligase SPL11 to the small GTPase OsRac1 for the regulation of PCD and innate immunity in rice.

## Results

### *Spin6* encodes a plant-specific RhoGAP protein

**Figure 1:**
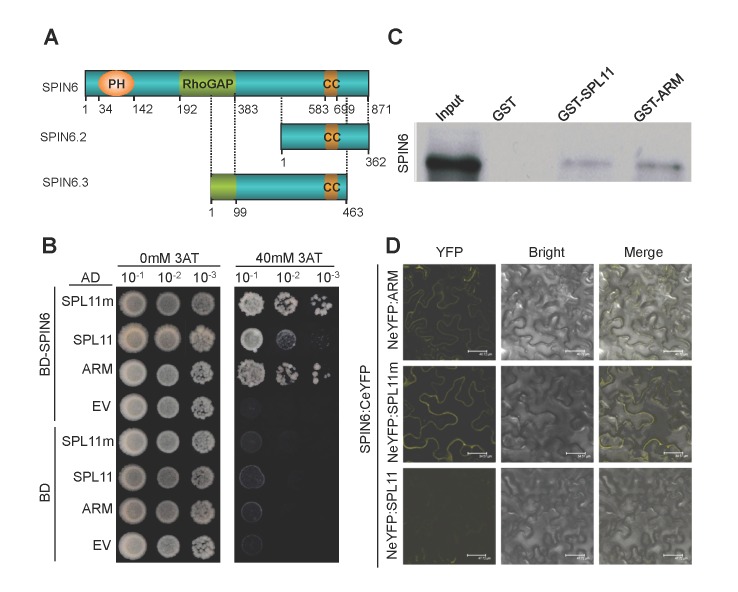
SPIN6 is a Rho GTPase-activating protein (RhoGAP) and interacts with SPL11 *in vitro* and *in vivo*. (A) The protein structure of SPIN6, SPIN6.2 and SPIN6.3. The position of the Pleckstrin Homology (PH) domain (34–142), RhoGAP domain (192–383), and coiled coil (CC) motif (583–699) are indicated. (B) SPIN6 interacts with SPL11 in yeast. SPL11 represents the full-length SPL11; ARM is the ARM domain of SPL11; and SPL11m is the SPL11 mutant with a three amino-acid deletion at C314P315T316 in the U-box domain, resulting in a loss-of-function of E3 ligase activity (Zeng et al., 2004). After they were diluted 10, 100, and 1000 times with sterilized-distilled H_2_O, the Mav203 yeast transformants were plated on synthetic dextrose medium without Trp, Leu, and His amino acids (SD-LTH) and with 0 mM or 40 mM 3-amino-1,2,4,-triazole (3AT), separately. (C) SPIN6 binds to SPL11 in the GST pull-down assay. His:SPIN6 and GST:SPL11, GST:ARM of SPL11 were used in the assay. (D) SPIN6 interacts with SPL11 in *N*. *benthamiana* in BiFC assay. SPIN6 was fused with the C-terminal of eYFP to make SPIN6:CeYFP. SPL11, ARM, and SPL11m were fused with the N-terminal eYFP to make NeYFP:SPL11, NeYFP:ARM, and NeYFP:SPL11m, respectively.

To search for novel components in the SPL11-mediated PCD and defense signaling pathway, we identified eight SPL11-interacting proteins (SPINs) in a yeast two-hybrid (Y2H) screen [24]. Among them, *Spin6* (Os07g46450) encodes a RhoGAP protein with a Pleckstrin Homology (PH) domain, a RhoGAP domain at the N-terminal and a C-terminal coiled coil (CC) motif (Fig. 1A). In a BLAST search of the rice genome using the *Spin6* sequence as a query, we found two PH/GAP-type SPIN6 homologs (Os03g11140 and Os03g24180) (Figure S1 in S1 Text). Sequence alignment analysis using both the cDNA and protein sequences of these genes showed that *Spin6* shares an identity of 61.4%, 81.7% at the nucleotide level and 40.7%, 79.7% at the protein level with Os03g11140 and Os03g24180, respectively (Table S1 and S2 in S2 Text). A protein sequence alignment analysis of the three rice genes with three Arabidopsis PH/RhoGAP orthologs revealed that the PH, GAP and CC domains in these proteins are highly conserved in both plants (Figure S2 in S1 Text).

In searching the rice genome databases, we found three *Spin6* putative splicing isoforms, i.e., Os07g46450.1/*Spin*6, Os07g46450.2/*Spin*6.2 and Os07g46450.3/*Spin*6.3. In this study, we used the longest isoform *Spin6* because *Spin6*.*2* lacks the PH and RhoGAP domains, and *Spin6*.*3* only contains a truncated RhoGAP domain and a truncated C-terminal (Fig. 1A). The alignment of the three protein sequences is shown in Figure S3 in S1 Text. Because *Spin6*.*2* and *Spin6*.*3* are unlikely to be functional rice Rho/GAP genes, we focused on the functional analysis of *Spin6* in this study.

A further analysis of the phylogenetic relationships among RhoGAP proteins in rice, *Arabidopsis*, human, and *Drosophila* revealed that RhoGAPs in rice and *Arabidopsis* can be assigned to two subgroups based on the composition of the N-terminal motif (Figure S4A in S1 Text) and that the evolutionary pathway of RhoGAPs has been quite different in plants than in humans and *Drosophila* (Figure S4B in S1 Text). These results suggested that SPIN6 belongs to the highly conserved and evolutionarily specific subfamily of plant RhoGAPs.

### SPIN6 interacts with SPL11 *in vitro* and *in vivo*

The interaction between SPIN6 and SPL11 in yeast was confirmed with a Y2H experiment with full-length SPIN6, full-length SPL11, the SPL11 ARM domain (ARM), and a truncated SPL11 with a three-amino-acid deletion at C^314^P^315^T^316^ in the U-box domain causing loss of E3 ligase activity (SPL11m) [7]. The Y2H assay showed that SPIN6 strongly interacted with the two truncated forms, ARM and SPL11m, but interacted only weakly with the full-length SPL11 (Fig. 1B). Further analysis of interacting domains in yeast revealed that the C-terminal of SPIN6 and the ARM domain of SPL11 are required for their interaction (Figure S5A and S5B in S1 Text). To determine the interaction specificity between SPIN6 and SPL11, we tested the interaction between SPL11 and the SPIN6 homolog RhoGAP protein Os03g24180 in yeast. The analysis showed that the two proteins did not interact (Figure S6A in S1 Text). Similarly, we obtained negative result in the Y2H assay between SPIN6 and the SPL11 homolog protein OsPUB12 (Os06g01304) (Figure S6B in S1 Text). These results demonstrated that the interaction between SPL11 and SPIN6 is specific.

**Figure 2:**
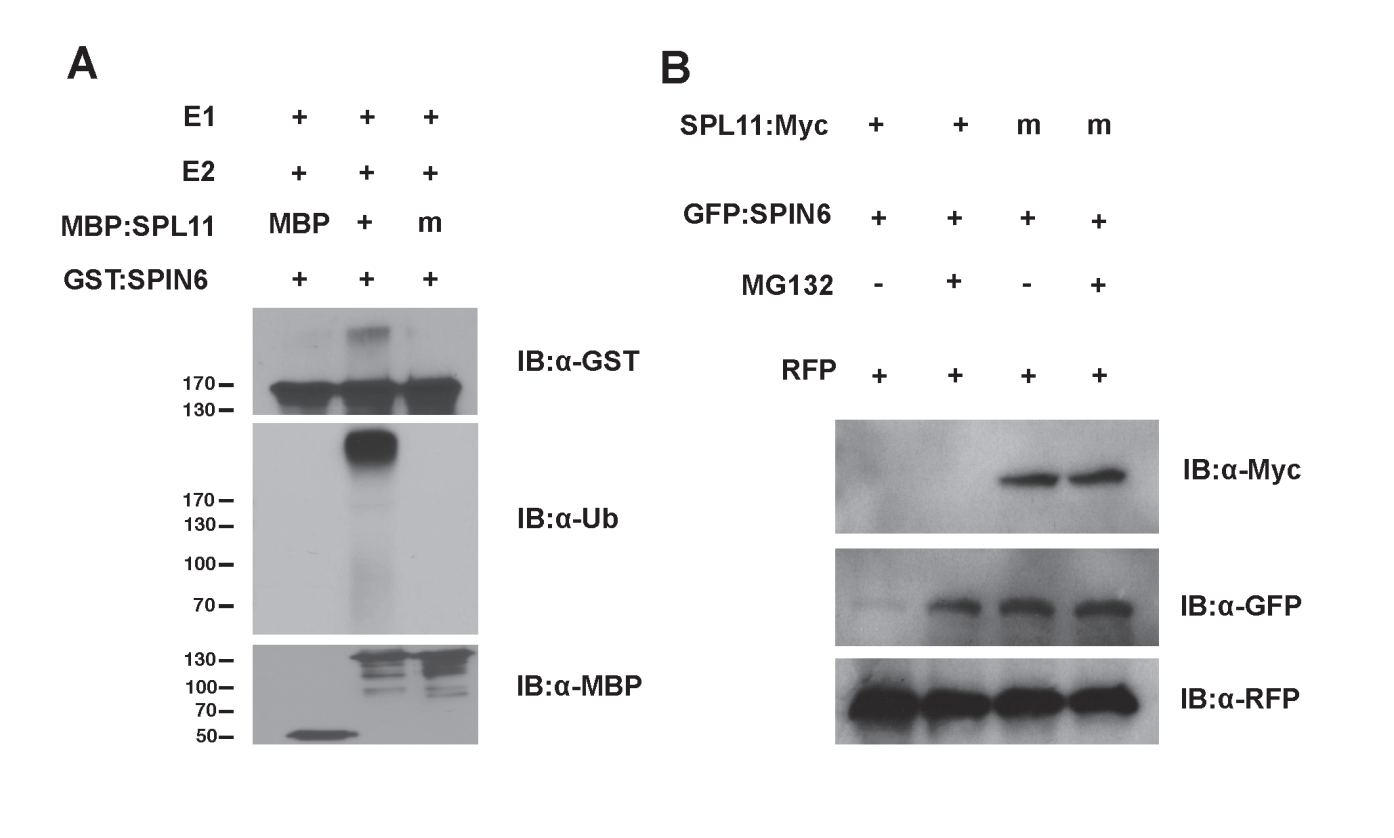
Ubiquitination of SPIN6 *in vitro* and degradation of SPIN6 in *N*. *benthamiana* by SPL11. **(A)** *In vitro* ubiquitination of SPIN6 by SPL11. The ubiquitination of GST:SPIN6 by MBP:SPL11 and the E3 ligase activity of SPL11 were detected by immunoblot with the anti-GST antibody and anti-ubiquitin antibody, respectively, in the presence of Arabidopsis E1 (At5g06460), E2 (AtUBC10, At5g53300) and ubiquitin. MBP and mutated SPL11 without E3 ligase activity (SPL11m) fused with MBP were used as the negative controls. Immunoblot with anti-MBP antibody was conducted as a loading control. **(B)** Degradation of SPIN6 promoted by SPL11 in *N*. *benthamiana*. Either SPL11:Myc or SPL11m:Myc was co-expressed with GFP:SPIN6 by agro-infiltration in *N*. *benthamiana*. Tissues were harvested at 3 days after the infiltration. MG132 (50μM), an inhibitor of 26S proteasome system, was infiltrated at 18 h before sampling with DMSO (-) as a control. RFP was expressed as an internal control.

To further confirm the interaction between SPIN6 and SPL11, we performed an *in vitro* GST pull-down assay. The results showed that both GST fusion SPL11 and ARM proteins bind to SPIN6 (Fig. 1C). Subsequently, a bimolecular fluorescence complementation (BiFC) assay in *N*. *benthamiana* revealed that SPIN6 interacts with SPL11 at the PM when leaves are co-infiltrated with the combinations of the *NeYFP*:*ARM* and *Spin6*:*CeYFP* plasmids or the *NeYFP*:*SPL11m* and *Spin6*:*CeYFP* plasmids (Fig. 1D, first and second panel, respectively). However, no signal was observed with the combination of the *NeYFP*:*Spl11* and *Spin6*:*CeYFP* plasmids, probably because the wild type (WT) SPL11 was not stable in *N*. *benthamiana* cells due to self-ubiquitination (Fig. 1D, third panel, also see results in Fig. 2B). The results of the control combinations are shown in Figure S7 in S1 Text. These results suggested that SPIN6 interacts with SPL11 *in vitro* and *in vivo*.

### SPL11 ubiquitinates SPIN6 *in vitro* and degrades SPIN6 through the 26S proteasome pathway *in planta*

To determine whether SPIN6 is a substrate of SPL11, we performed an *in vitro* ubiquitination assay that including the following purified proteins of E1, E2, MBP:SPL11, MBP:SPL11m, the E3 ligase dead mutant [7], and GST:SPIN6 were included. The E3 ligase activity of the wild-type SPL11 and SPL11m were confirmed by the immunoblot analysis with the anti-ubiquitin antibody (Fig. 2A, second and third lane, respectively, in the second panel). Interestingly, high molecular weight bands were only observed in the reaction with MBP:SPL11 but not with MBP:SPL11m in the immunoblot with the anti-GST antibody (Fig. 2A, second and third lane, respectively, in the first panel). To further confirm the ubiquitination result, we washed the GST:SPIN6 beads with the PBST solution (Phosphate Buffered Saline with 0.5% Triton X-100, [7]) after the E3 ligase reaction and performed an immunoblot analysis either with anti-ubiquitin or anti-GST antibody. The analysis with the anti-GST antibody showed that the high molecular weight bands above GST:SPIN6 were only present in the reaction with the wild type SPL11 but not with SPL11m (Figure S8A in S1 Text, second lane in the first panel) and that the ubiquitinated GST:SPIN6 was only detected by the anti-ubiquitin antibody only in the reaction with the wild-type SPL11 (Figure S8A in S1 Text, second lane in the second panel). These results clearly indicate that SPIN6 is ubiquitinated by SPL11 *in vitro*.

To investigate whether the ubiquitination of SPIN6 by SPL11 can lead to instability of SPIN6 *in vivo*, we co-infiltrated different combinations of agrobacteria carrying the *Spl11*:*Myc* and *GFP*:*Spin6* plasmids into *N*. *benthamiana* leaves with or without the 26S proteasome inhibitor MG132. Total protein was extracted 3 days after the infiltration, and MG132 was infiltrated into the same leaves 18 h before tissue collection. The immunoblot analysis revealed that the GFP:SPIN6 was degraded by SPL11:Myc (Fig. 2B, lane 1 in the second panel) but that the SPIN6 degradation was inhibited by MG132 (Fig. 2B, lane 2 in the second panel). In contrast, SPIN6 was not degraded when the mutated *Spl11m*:*Myc* plasmid without E3 ligase activity was expressed with or without the MG132 treatment (Fig. 2B, lane 3 and 4 in the second panel). The SPL11:Myc fusion protein was not stable in *N*. *benthamiana* with or without MG132 treatment (Fig. 2B, lane 1 and 2 in the first panel), probably due to self-ubiquitination. However, the SPL11 fusion protein was slightly visible when the immunoblot was overexposed for 15 min (Figure S8B in S1 Text, second lane in the first panel) and it was clearly visible for over 20 min (Figure S8B in S1 Text, second lane in the second panel).

### Knock-down and out of *Spin6* transcripts causes PCD and enhances the resistance to the rice blast pathogen and the bacterial blight pathogen

**Figure 3:**
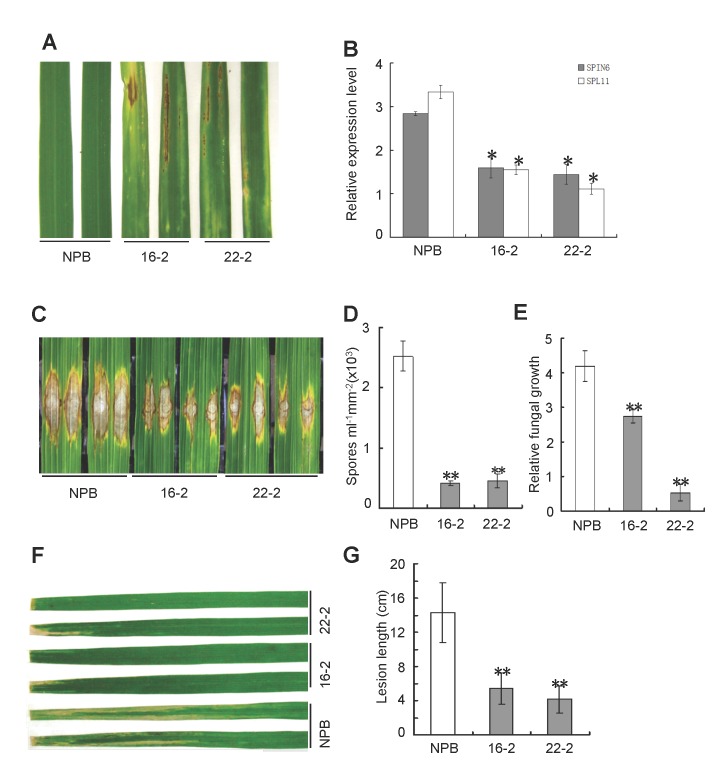
Knock-down of *Spin6* results in cell death phenotypes and enhanced resistance to the rice blast pathogen *M*. *oryzae* and the bacterial blight pathogen *Xoo* in rice. **(A)** Cell death phenotypes of the *Spin6* RNAi silencing lines. Japonica rice Nipponbare (NPB) is the wild type, and 16–2 and 22–2 are *Spin6* RNAi lines. **(B)** The relative expression level of *Spin6* and *Spl11* in the wild type and *Spin6* RNAi lines as determined by real-time quantitative PCR. The data were normalized with ubiquitin. **(C)** Lesion-size of the *Spin6* RNAi lines and the wild type plants inoculated with *M*. *oryzae* isolate RO1–1. **(D)** Number of *M*. *oryzae* spores produced by lesions on the *Spin6* RNAi lines and the wild type. **(E)** Relative *M*. *oryzae* biomass in lesions in the *Spin6* RNAi lines and the wild type. **(F)** Lesion-size of the *Spin6* RNAi lines and the wild type infected with *Xoo* strain race 6. **(G)** Bacterial blight lesion length in the *Spin6* RNAi lines and the wild type. Values are means and standard errors of three replications. Significance was determined at *P<0.05 and **P<0.01 (n≥3) with a *t*-test.

To understand the biological function of *Spin6*, we made an RNAi construct that targets the 302-bp 3’ UTR region in *Spin6* (Figure S9A and B in S1 Text). To search possible similar sequences in the rice genome, we performed a whole genome similarity search with the 302-bp silencing fragment using the Mega BLAST program at the NCBI website. The analysis showed that this fragment only specifically targets the *Spin6* gene (Figure S10A in S1 Text). Further, a BLASTN search using the RNAi fragment indicated that all of the possible 21-bp nucleotides to be generated from the RNAi sequence only target to the *Spin6* sequence with 100% matches (Figure S10B in S1 Text). The *Spin6*-silencing transgenic rice was generated by transforming the RNAi construct in the calli of Nipponbare (NPB) via *Agrobacterium*-mediated transformation. Over 20 independently transformed lines were obtained in the T0 generation. The presence of the RNAi transgene and the hygromycin gene was determined in both the T_0_ and T_1_ generation by PCR. We identified six homozygous lines that had a single transgene insertion in the genome and that showed cell death phenotypes in the greenhouse. To confirm the cell death phenotypes, we grew two homozygous lines of the T3 generation (16–2 and 22–2) in a growth chamber under normal growth conditions (see details in Experimental Procedures). About 4 weeks after planting, obvious cell death-like lesions were evident in both RNAi lines but not in the NPB plants (Fig. 3A, Figure S11A and B in S1 Text). The expression level of *Spin6* was significantly lower in these two lines than in NPB (Fig. 3B). The cell death phenotype was confirmed in the next three generations. Intriguingly, the transcription level of *Spl11* was also significantly suppressed in the *Spin6* RNAi lines (Fig. 3B), indicating that the suppression of *Spin6* transcripts might have a feedback effect on the expression of *Spl11*. Because knock-out of *Spl11* in rice enhances non-race-specific resistance to both *M*. *oryzae* and *Xoo* [7], we also evaluated the resistance of *Spin6* RNAi lines to both pathogens. When rice leaves were inoculated with the compatible *M*. *oryzae* isolate RO1–1 by the punch inoculation method [25], the lesion size, spore number, and relative fungal biomass in the lesions were significantly lower in the two *Spin6* RNAi lines (16–2 and 22–2) than in NPB (Fig. 3C, D and E). Similarly, lesions caused by *Xoo* strain race 6 were much shorter in the *Spin6* RNAi lines than in NPB (Fig. 3F and G).

To further confirm the phenotype of the *Spin6* RNAi lines, we obtained a *Spin6* T-DNA insertion mutant from Korea [26]. PCR-based genotyping analysis indicated that the T-DNA fragment was inserted into the C-terminal of the 13^th^ intron of the *Spin6* gene (Figure S12A and B in S1 Text), and the transcription level of *Spin6* was significantly reduced (Figure S12C in S1 Text). Strikingly, *spin6* displayed much more severe cell death phenotypes than to the RNAi lines (Figure S13A in S1 Text) and no seeds or few seeds were harvested from the mutant plants under high humidity and strong sunlight conditions. We evaluated the resistance phenotype of the mutant to *M*. *oryzae* and *Xoo* in growth chamber conditions. Like the *Spin6* RNAi plants, the *spin6* mutant plants showed enhanced resistance to *M*. *oryzae* isolate RB22 (Figure S13B in S1 Text); when inoculated with RB22, *spin6* plants supported lower *M*. *oryzae* spore production (Figure S13C in S1 Text) and lower fungal biomass than the wild-type Hwayoung (Figure S13D in S1 Text). Similarly, lesions were much shorter on *spin6* than on the wild-type Hwayoung when the plants were inoculated with *Xoo* race RB6 (Figure S13E in S1 Text). Taken together, these results suggested that *Spin6* negatively regulates both plant cell death and disease resistance to fungal and bacterial pathogens in rice.

### Knock-down and-out of *Spin6* elevates both chitin- and flg22-mediated defense signaling in rice

**Figure 4:**
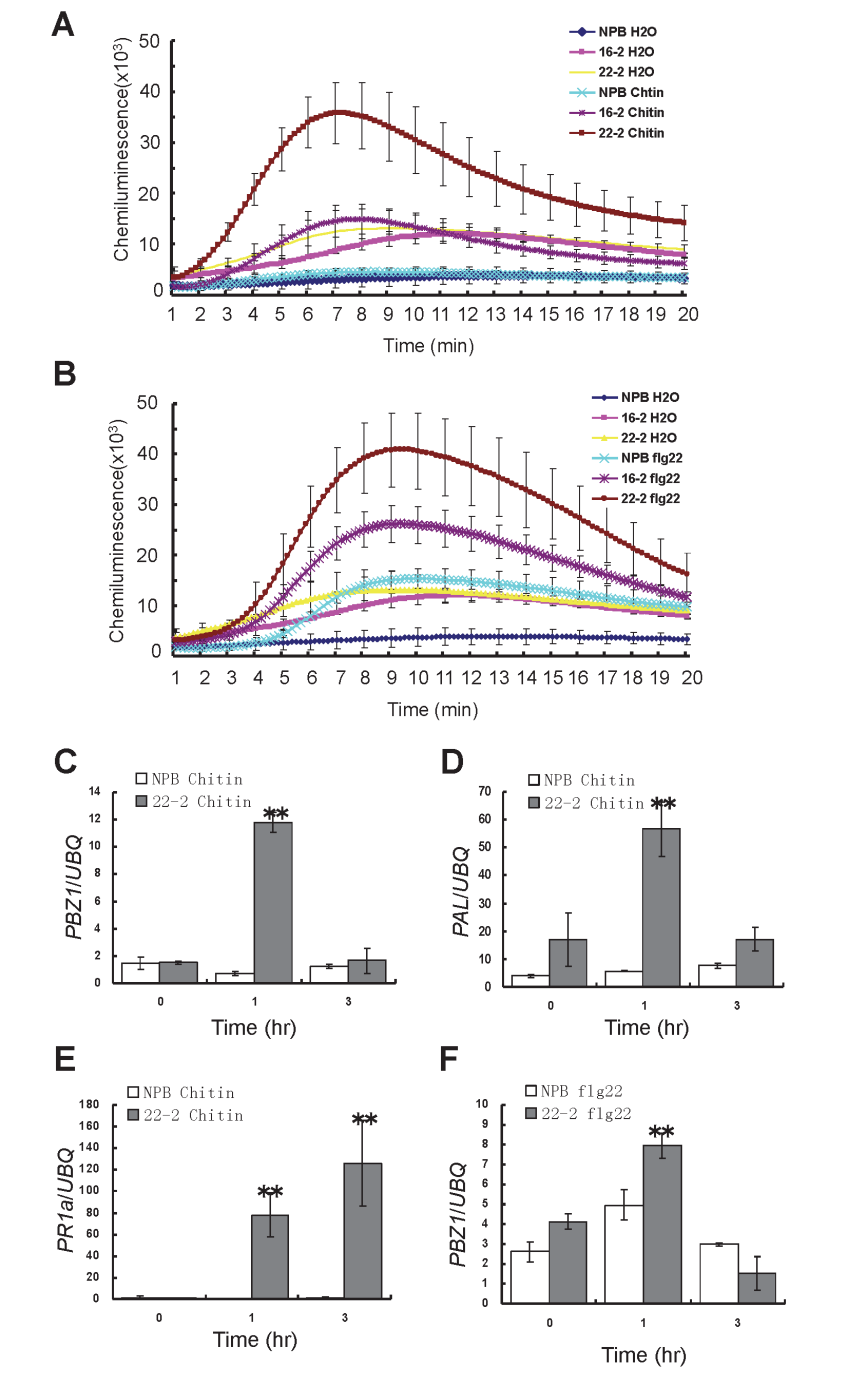
ROS generation and defense-related gene expression of the *Spin6* RNAi lines after chitin and flg22 treatments. (A), (B) ROS accumulation dynamics of the *Spin6* RNAi lines (16–2 and 22–2) and the wild type NPB after the chitin or flg22 treatment. Water served as the control. (C)-(F) The relative expression level of defense-related genes *PBZ1* (C, F), *PAL* (D), and *PR1a* (E) in the *Spin6* RNAi line 22–2 and NPB after treatment with chitin and flg22. Values are means and standard errors of three replications. Significance was determined level at *P<0.05 and **P<0.01 (n = 3) with a *t*-test.

To further investigate which pathways are involved in the *Spin6*-mediated defense signaling pathway, we used chemical luminescence to monitor the dynamics of ROS generation in the *Spin6* RNAi plants treated with the PAMPs chitin and flg22, these PAMPs trigger PRR OsCEBiP/OsCERK1- and OsFLS2-mediated PTI signaling in rice [25,27]. The results revealed that ROS accumulation was significantly higher in the two *Spin6* RNAi lines than in NPB in response to both chitin and flg22 treatments; the peak ROS level, which occurred about 10 min after the treatments were applied, was two- to four-times greater in the *Spin6* lines than in NPB (Fig. 4A and B). Even in the water control, the basal ROS level was three-times higher in the *Spin6* RNAi lines than in NPB (Fig. 4A and B). This finding was confirmed by quantification of the endogenous H_2_O_2_ level, which in the water control was about two-times greater in the *Spin6* lines than in NPB (Figure S14A in S1 Text). Similarly, the *spin6* mutant also showed a higher ROS accumulation (Figure S13F and G in S1 Text) and endogenous H_2_O_2_ level (Figure S14B in S1 Text) in response to both chitin and flg22 treatments comparing to the wild type. Therefore, these results indicated that silencing and knock-out of *Spin6* in rice enhances chitin- and flg22-triggered ROS accumulation.

To determine whether *Spin6* suppression alters the expression of defense genes, we analyzed the expression patterns of three defense-related genes (*PBZ1*, *PAL*, and *PR1a*) in the *Spin6* RNAi line 22–2 and in NPB after treatment with chitin and flg22. At 1 h after chitin treatment, the expression of the genes *PBZ1* and *PAL* was > 10-times greater in the *Spin6* RNAi plants than in NPB, but the expression returned to the normal level in the RNAi plants at 3 h after chitin treatment (Fig. 4C and D). At 1 and 3 h after chitin treatment, *PR1a* expression was more than 80 times in the *Spin6* RNAi line than that in NPB (Fig. 4E). Conversely, only *PBZ1* was significantly induced in the RNAi line relative to NPB at 1 h after flg22 treatment (Fig. 4F). The expression of both *PAL* and *PR1a* was not significantly induced by the flg22 treatment in either the RNAi line or NPB (Figure S15A and B in S1 Text). Together, these results suggested that *Spin6* plays a negative role in the regulation of PTI by suppressing ROS generation and defense gene signaling in rice.

In addition, we investigated the expression pattern of *Spin6*- and defense-related genes in the *Spin6* RNAi and wild type NPB plants before and after blast inoculation. Before inoculation, the expression of *Spin6* and *Spl11* was significantly lower and the expression of *RbohB* was slightly low in the *Spin6* RNAi plants than that in NPB plants (Figure S16A, B and C in S1 Text). The expression of other five genes, i.e., *OsRac1*, *PR1a*, *OsNAC4*, *PR5* and *PBZ1* was the same in both genotypes (Figure S16E, F, G and H in S1 Text). At 24 h after blast inoculation, the expression of *Spl11* was induced but the expression of *Spin6* and *OsRac1* was reduced in both genotypes. Interestingly, except for the highly induced expression of *OsNAC4* in both genotypes, the expression of the three defense-related genes, *PR1*a, *PR5* and *PBZ1*, was highly induced only in the *Spin6* RNAi plants at 24 h after inoculation (Figure S16E, F, G and H in S1 Text). These results provided further evidence for the enhanced resistance in *Spin6* RNAi plants against *M*. *oryzae* as shown in Fig. 3A.

### SPIN6 interacts with the small GTPase OsRac1, catalyzes the GTP-bound OsRac1 to the GDP-bound state *in vitro* and inactivates OsRac1 in rice protoplasts

**Figure 5:**
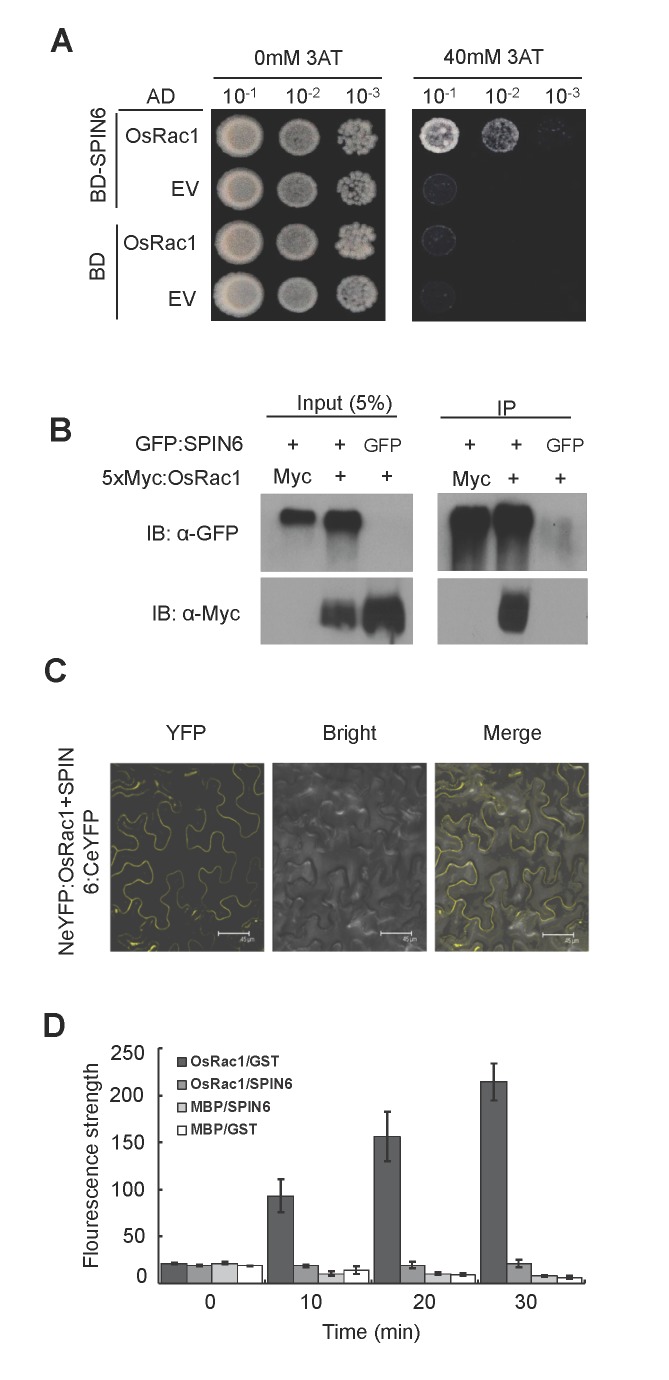
SPIN6 interacts with OsRac1 *in vitro* and *in vivo*, and catalyzes OsRac1 hydrolysis *in vitro*. **(A)** SPIN6 interacts with OsRac1 in yeast. The Mav203 yeast transformant diluted 10, 100, or 1000 times was plated on synthetic dextrose medium without Trp, Leu, or His (SD-LTH) and with 0 mM or 40 mM 3-amino-1,2,4,- triazole (3AT). EV-Empty Vector (**B**) The Co-IP assay of SPIN6 and OsRac1 in *N*. *benthamiana*. The *GFP*:*Spin6* and *Myc*:*OsRac1* plasmids were used for co-infiltration in *N*. *benthamiana*. (**C**) BiFC assay of SPIN6 and OsRac1 in *N*. *benthamiana*. The *Spin6*:*CeYFP* and *NeYFP*:*OsRac1* plasmids were used for co-infiltration. (**D**) *In vitro* GAP activity assay of SPIN6. SPIN6 refers to GST-fused SPIN6 protein. OsRac1 refers to MBP-fused OsRac1 protein. GST and MBP are controls. Values are means and standard errors of three replications.

Because RhoGAPs can facilitate small GTPases hydrolysis, we speculated that the RhoGAP SPIN6 might target some Rac proteins in rice. Among the seven OsRacs, OsRac1 is a crucial component in the regulation of plant cell death and innate immunity [21]. We analyzed the expression patterns of three components in the OsRac1-mediated complex: *OsRac1*, *OsSGT1*, and *OsRAR1*. The qRT-PCR analysis revealed that, after both chitin and flg22 treatments, the expression of all three genes was significantly up-regulated in the *Spin6* RNAi plants relative to NPB (Figure S15C-H in S1 Text), suggesting a possible relationship between *Spin6* and *OsRac1*. Then, we performed a Y2H experiment to determine whether SPIN6 can interact with OsRac1. The assay showed that SPIN6 interacts with OsRac1 in yeast (Fig. 5A). This interaction was further confirmed by both Co-IP and BiFC assays in *N*. *benthamiana*. The Co-IP assay showed that GFP:SPIN6 can immunoprecipitate Myc:Rac1, while neither GFP nor Myc controls were able to immunoprecipitate OsRac1 or SPIN6 (Fig. 5B). In the BiFC assay, a yellow fluorescence at the PM of leaf cells was detected when the *Spin6*:*CeYFP* and *NeYFP*:*OsRac1* plasmids but not the control combinations were co-expressed in *N*. *benthamiana* (Fig. 5C; Figure S6 in S1 Text). Together, these results suggested that SPIN6 interacts with OsRac1 *in vitro* and *in vivo*.

To determine whether SPIN6 possesses the RhoGAP activity that facilitate the hydrolysis of the GTP-bound OsRac1, we performed an *in vitro* RhoGAP activity assay. The result showed that the fluorescence signal in the reaction containing the OsRac1 and SPIN6 proteins was as low as that in the two negative controls because the fluorescent GTP-bound OsRac1 was hydrolyzed to GDP-bound forms in the presence of the SPIN6 protein (Fig. 5D). In contrast, the fluorescence signal in the reaction with the OsRac1 and GST proteins increased rapidly 10 min after the reaction began. No fluorescence signal was observed in the MBP and SPIN6 or MBP and GST combinations. These results suggested that SPIN6 can catalyze the GTP-bound OsRac1 into the GDP-bound inactive form of OsRac1.

**Figure 6:**
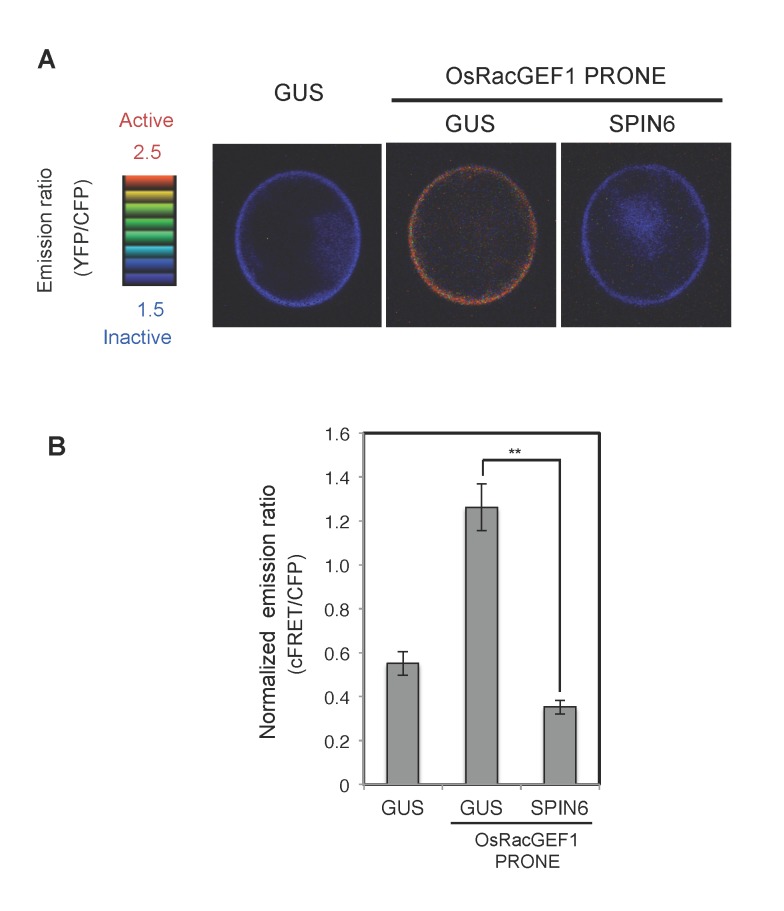
Analysis of RhoGAP activity of SPIN6 toward OsRac1 in rice protoplasts. **(A)** Images of confocal laser-scanning micrographs of rice protoplasts expressing the *Raichu-OsRac1* constructs. Rice protoplasts were co-transfected with constructs expressing *Raichu-OsRac1* and individual constructs. The FRET images are shown in intensity-modulated display mode, which associates color hue with emission ratio values and the intensity of each hue with the brightness of the source image. **(B)** Normalized emission ratio of cFRET/CFP. Double asterisks indicate significant differences (P < 0.01). Error bars indicate SE (N≧30).

To monitor *in vivo* GAP activity of SPIN6 toward OsRac1, we used the FRET sensor called Raichu-OsRac1 [21]. The YFP/CFP fluorescence ratio of Raichu-OsRac1 provides an estimate of the activation state of OsRac1 *in vivo*, with low and high ratios of YFP/CFP fluorescence corresponding to low and high levels of OsRac1 activation, respectively. Using Raichu-OsRac1, we monitored the activation level of OsRac1 in the presence of SPIN6 *in vivo*. Because the basal activation level of OsRac1 was very low (Fig. 6A, left photo), thus, we expressed the PRONE domain of OsRac1GEF1 (OsRacGEF1 PRONE) that activates OsRac1 in rice cells [23]. The ratio of YFP/CFP fluorescence was significantly higher in the cells expressing *OsRacGEF1 PRONE* than in the cells expressing the control GUS vector, which showed that OsRacGEF1 PRONE activates OsRac1 in rice protoplasts (Fig. 6A, middle photograph). In contrast, this activation was remarkably suppressed by the expression of SPIN6 (Fig. 6A, right photograph). The normalized emission ratio (cFRET/CFP) was much lower in the rice protoplasts transfected with SPIN6 than that in the GUS control (Fig. 6B). Taken together these results provided evidence that SPIN6 has GAP activity towards OsRac1 in rice protoplasts.

## Discussion

### SPIN6 negatively regulates plant cell death and innate immunity by associating with SPL11 in rice

The rice U-box E3 ligase SPL11 and its *Arabidopsis* ortholog PUB13 play important roles in the regulation of both defense and flowering [7,10,11]. Previously, we identified eight *Spin* genes from the rice cDNA library in a Y2H screen [24]. Among them, SPIN1 is involved in flowering-time regulation. However, the component that transduces the SPL11 signal for PCD and defense response signaling was unknown before the current study. In this study, we found that SPL11 interacts with the RhoGAP SPIN6 *in vitro* and *in vivo* and ubiquitinates SPIN6 *in vitro*. Co-infiltration assays in *N*. *benthamiana* showed that SPL11 degrades SPIN6 via the 26S proteasome-dependent pathway. Silencing of *Spin6* enhances PCD and non-race-specific resistance to both the rice blast and the bacterial blight pathogens and results in phenotypes similar to those in the *spl11* mutant. We also found that *Spl11* transcription is significantly down-regulated in *Spin6* RNAi lines, indicating a feedback regulation of *Spin6* over *Spl11*. These results suggest that SPIN6 negatively regulates PCD and disease defense by directly associating with SPL11. In addition, the results from the Co-IP and hydrolysis activity assays demonstrated that SPIN6 interacts with OsRac1 and can catalyze GTP-bound OsRac1 into the GDP-bound state *in vivo*. The FRET analysis in rice protoplasts demonstrated that SPIN6 has *in vivo* GAP activity towards OsRac1. Our study has provided clear evidence for the linkage between a U-box E3 ligase-mediated ubiquitination pathway and a small GTPase-associated defensome system. Such a linkage has not been previously reported in any plant.

Although RhoGAPs are known to have critical roles in diverse cell processes [13], there are only few reports on the function of these proteins in plant immunity, and these include RanGAP2 in *N*. *benthamiana* [15,16], MAGAP1 in barley [17], and OsGAP1 in rice [18,19]. How these proteins are regulated in plant immune responses is largely unknown. In this study, we found that the RhoGAP SPIN6 is ubiquitinated and degraded by SPL11 via the 26S proteasome pathway *in planta*. The E3 ligase activity of SPL11 is required for the degradation. Because the C-terminal of SPIN6 is involved in the interaction with SPL11 in yeast, we speculate that SPL11 ubiquitinates the SPIN6 C-terminal region. It seems that SPIN6 is poly-ubiquitinated by SPL11 because many high molecular weight bands above the SPIN6 band were observed the *in vitro* ubiquitination assays. In addition, we found that the *Spl11* transcript level is lower in the *Spin6* RNAi plants than in NPB, suggesting that down-regulation of *Spin6* also suppresses *Spl11* expression probably due to an unknown feedback regulation. When the SPL11 and SPIN6 antibodies become available, it will be interesting to determine the relationship between SPL11 and SPIN6 at the protein level.

Additionally, the *Spl11* gene was demonstrated to be involved in flowering time regulation through mono-ubiqutinating the RNA-bing protein SPIN1 [24]. We also found that the *Spin6* RNAi plants showed delayed flowering time under both short day (SD) and long day (LD) conditions (Figure S17A and B in S1 Text). We checked the expression of the three flowering time-related marker genes; *OsGI*, *Hd1*, *Hd3a*, we found that the *OsGI* and *Hd3a* are suppressed in RNAi lines under both SD and LD conditions, but for *Hd1*, it is only suppressed in SD condition (Figure S17C, D and E in S1 Text). The function of *Spin6* in flowering time regulation will be further investigated.

### SPIN6 is involved in chitin- and flg22-triggered PTI signaling in rice

Our current data show that ROS levels are significantly up-regulated in both *Spin6* RNAi and T-DNA insertion mutant plants after chitin and flg22 treatments. Even without any treatment, the basal ROS level and endogenous H_2_O_2_ content are higher in the *Spin6* RNAi or mutant plants than in the wild type. This is consistent with previous reports of the high level of ROS and a rapid ROS burst in the *spl11* mutant after treatment with an elicitor from the rice blast fungus [8,9]. In addition, the analysis of defense-related gene expression showed that *PBZ1* is highly up-regulated after both chitin and flg22 treatments, while *PAL* and *PR1a* were up-regulated only after chitin treatment. These results suggested that the chitin- and flg22-triggered PTI signaling involves in SPIN6-meidated defense signaling regulation. However, what components are involved in *Spin6*-mediated defense signaling in rice requires further investigation

A recent study showed that OsRacGEF1 acts as a guanine nucleotide exchange factor for OsRac1 [23]. OsRacGEF1 interacts with the chitin co-receptor OsCERK1, and the activated OsRacGEF1 is required for chitin-driven immune responses and resistance to *M*. *oryzae*. Because SPIN6 is also involved in chitin-triggered immunity, the relationship between SPIN6 and OsCERK1 and OsRacGEF1 during rice immunity warrants investigation

### SPIN6 negatively modulates the OsRac1-mediated immune signaling in rice

RhoGAP proteins regulate small GTPases by altering their GTP state [13]. In our study, we found that SPIN6 interacts with the rice small GTPase OsRac1 and has RhoGAP activity in that it catalyze OsRac1 hydrolysis. In addition, the transcription level of *OsRac1* is highly induced in the *Spin6* RNAi plants after chitin and flg22 treatment, and the expression of two major components in the OsRac1 complex, *OsSGT1* and *OsRAR1*, is significantly up-regulated. These results indicate that SPIN6 plays an important role in regulating *OsRac1*-dependent immune signaling in rice. As shown in previous studies, OsRac1 is a key signaling integrator for both PTI and ETI pathways and interacts with OsRBOH to transduce cell death and defense signaling in rice [20]. One important function of SPIN6 might be to maintain an optimum level of active OsRac1 so that all biological processes regulated by OsRac1 in rice cells are well controlled and balanced. A recent study found that OsRacGEF1 activates inactive forms of OsRac1 and is a positive regulator of rice immunity [23]. It will be important to determine the relationship between SPIN6 and OsRacGEF1 in the control of OsRac1 and to determine whether OsRac1, OsRacGEF1, and SPIN6 form a protein complex to regulate defense responses in rice. Furthermore, because OsRacGEF1 is phosphorylated by OsCERK1 [23], future research should determine whether SPIN6 can also be phosphorylated by OsCERK1 and how SPIN6 contributes to chitin-mediated immunity.

### A working model to illustrate the relationships among SPL11, SPIN6, and OsRac1

**Figure 7:**
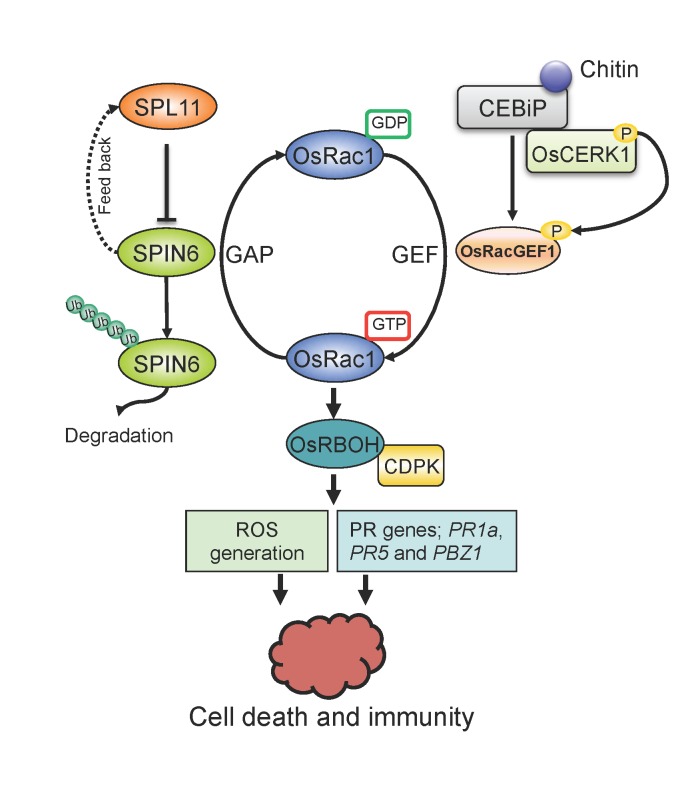
Proposed working model of the relationship between SPL11, SPIN6, and OsRac1. By associating with RhoGAP protein SPIN6, the E3 ligase SPL11 negatively modulates OsRac1-mediated immune signaling. SPIN6 is ubiquitinated and degraded by the E3 ligase SPL11 via the 26S proteasome system. From the GDP state to the GTP state, OsRac1 is activated by the GEF protein OsRacGEF1, then associates with the NADPH oxidases OsRBOH/CDPK complex to trigger ROS generation. The activation of OsRac1 requires the phosphorylation by the kinase protein OsCERK1, a co-receptor of the PAMP effector chitin. OsCERK1 dimerizes with LysM protein CEBiP1 to perceive chitin signaling. The interaction between SPIN6 and OsRac1 may lead to the change of OsRac1 from the GTP state to the GDP state, which reduces the active form of OsRac1 in rice cells. Mutation in the *Spin6* gene may cause accumulation of ROS and PR proteins, i.e., PR1a, PR5 and PBZ1, that results in plant cell death and immunity.

Over the last 10 years, our laboratory has obtained considerable information on the function of the rice U-box E3 ligase SPL11 in the regulation of PCD and defense. We previously found that SPL11 is a functional U-box E3 ligase and negatively regulates PCD and defense. In this study, we found that SPL11 ubiquitinates the RhoGAP SPIN6 and degrades it through the 26S proteasome-mediated pathway. We also found that *Spin6* is a negative regulator of PCD and defense because the expression of the three defense-related genes, *PR1*a, *PR5* and *PBZ1*, is highly induced in the *Spin6* RNAi plants after blast inoculation, consistent with their enhanced resistance to *M*. *oryzae*. *In vitro* and *in vivo* GAP analysis showed that SPIN6 is the RhoGAP protein of OsRac1 that can switch OsRac1 from active forms to inactive forms. Knocking out or down of the *Spin6* gene may lead to the accumulation of active forms of OsRac1, which causes ROS generation, defense activation and cell death. Because OsRac1 is a signal integrator of the PAMP elicitor chitin, which is perceived by PRRs like OsCERK1, the SPL11-SPIN6-mediated pathway might connect with the chitin-mediated pathway to regulate PCD and immune responses. Based on previously published results and the new results from this study, we propose a working model to illustrate the relationships among SPL11, SPIN6, and OsRac1 in rice immunity (Fig. 7).

## Materials and Methods

### Plant materials and rice transformation

Japonica cultivar Nipponbare (NPB) was used for rice transformation. Rice seeds were germinated as previously described [28]. After germination, rice seedlings were transferred to soil and grown in a growth chamber at 22°C, 80% relative humidity (RH), and a 12-h/12-h light/dark photoperiod. *N*. *benthamiana* seeds were germinated in the same manner as rice seeds. After germination, *N*. *benthamiana* seedlings were transferred to soil and grown in a growth chamber at 22°C, 70% RH, and a 16-h/8-h light/dark photoperiod. *N*. *benthamiana* plants were used for agroinfiltration when they were 4 to 7 weeks old.

The construct pANDA-SPIN6RNAi was made for the generation of SPIN6 RNAi transgenic rice (Figure S9A in S1 Text) [29]. *Agrobacterium*-mediated transformation was used to generate the transgenic rice plants as described previously [30]. All genes accession and primer pairs used in this study are listed in *SI data* (Table S3 in S2 Text).

### *M*. *oryzae* and *Xoo* inoculation

A punch inoculation method was used to inoculate rice plants with *M*. *oryzae* isolate RO1–1 as described by [25]. Briefly, leaves of 5- to 8-week-old rice plants were wounded with a hole-punch (one leaf per plant), and 5 μl of an *M*. *oryzae* spore suspension (5x10^5^ spores ml^-1^) was applied to the injured area, which was sealed with cellophane tape. The inoculated plants were kept in darkness at 80% RH for 24 h before they were transferred to a growth chamber at 28°C, 80% RH, and a 12-h/12-h light/dark photoperiod. After the plants had been in the growth chamber for 10 days, lesion size, spore number per lesion, and fungal biomass per lesion were determined. A leaf-clipping method was used to inoculate rice plants at the booting stage with *Xoo* isolate race 6 as previously described [31]. The *Xoo*-inoculated plants were maintained as described for *M*. *oryzae*-inoculated plants. Lesion lengths were measured 14 days after inoculation.

### RNA isolation and quantitative RT-PCR

RNA was extracted from rice leaves with Trizol reagent (Invitrogen) and was treated with Dnase I. A 1-μg quantity of RNA in a 20-ul reaction volume was used for cDNA synthesis using the Promega reverse transcription system. After the cDNA was diluted 5–10 times, 1 μl of the diluted cDNA in a 25-μl reaction volume with BioRad SYBR supermix buffer was used for real-time quantitative PCR with a BioRAD iQ2 machine and the following program: 40 cycles at 95°C for 15 s, 60°C for 30 s, and 72°C for 20 s. Three replications were performed. The data were normalized with ubiquitin, and the means of three replications are presented.

### ROS measurement

ROS was detected as previously described [25]. Briefly, leaves were cut into pieces (0.25 cm^2^) and submerged in distilled water for 3 h. Three leaf pieces were then placed in a 1.5-ml microcentrifuge tube with 100 μl of luminol (Bio-Rad Immun-Star horseradish peroxidase substrate 170–5040), 1 μl of horseradish peroxidase (Jackson ImmunoResearch), and 100 nM flg22 or 8 nM hexa-N-acetyl-chitohexaose, or distilled water as a control. The samples were immediately placed in a Glomax 20/20 luminometer (Promega), and luminescence was measured at 10-s intervals for 20 min.

### Yeast two-hybrid assay

The ProQuest yeast two-hybrid system (Invitrogen) was used according to the manufacturer’s protocol. SPL11-interacting genes were screened as previously described [24]. For interaction confirmation, the cDNAs of the genes *Spl11*, *Spin6*, and *OsRac1* were separately cloned into the BD vector pDBLeu or the AD vector pPC86. Corresponding BD and AD constructs were then co-transformed into the yeast strain Mav203 and selected on synthetic dextrose medium without Trp or Leu (SD-Leu-Trp). The single transformant was picked up and diluted 10, 100, or 1000 times in sterilized-distilled water. For the interaction assay, 1 μl of each dilution was plated on SD-Leu-Trp-His medium with 0 mM or 40 mM 3-amino-1,2,4,-triazole (3AT).

### GST pull-down

The full-length *Spin6* cDNA was cloned into the vector pET28a for *in vitro* transcription/translation of SPIN6 protein using the TNT rabbit reticulocyte lysate translation system (Promega). The full-length *Spll11* cDNA and the *Spl11* ARM domain fragment were cloned into the vector pGEX-6p-1, and the GST:SPL11 fusion proteins were expressed and purified according to the manufacturer’s instructions (Amersham Biosciences). GST pull-down was performed as previously described [24]. Bound SPIN6 was detected by the streptavidin–horseradish peroxidase chemiluminescent method using the Transcend Nonradioactive Detection System (Promega).

### *Agrobacterium*-mediated transient expression in *N*. *benthamiana*

Agroinfiltration was performed as described [32]. Briefly, *Agrobacterium* strain GV3101 with corresponding constructs was incubated at 28°C with shaking (220 rpm) for 18 h. Bacteria were collected by centrifugation at 4000 rpm for 10 min and were resuspended to a final OD_600_ of 1.5 in MES buffer (10 mM MgCl_2_ and 10 mM MES, pH 5.6) with 150 nM acetosyringone. After 3 h, the suspensions were infiltrated into *N*. *benthamiana* leaves. After 3 days, leaf samples were collected for protein extraction or microscopic observation.

### BiFC and co-IP analysis

Full-length cDNAs of *Spin6*, *Spl11*, and *OsRac1* were separately cloned into vectors pSPYNE(R)173 and pSPYCE(M) for BiFC assay [33]. The constructs were expressed in *N*. *benthamiana* by the agroinfiltration method. A Leica confocal microscope was used for YFP fluorescence observation with excitation at 515 nm and emission at 525 nM; images were captured with a Leica Microsystems camera (Leica, Heidelberg GmbH).

For Co-IP, GFP:SPIN6 and Myc:OsRac1 protein was isolated from *N*. *benthamiana* leaves using a native buffer as previously described [34] Co-IP was performed as previously described [25]. Briefly, 1 ml of protein solution mixed with 15 μl of anti-GFP antibody (Roche) was gently shaken at 4°C for 4 h. The samples were then mixed with 10 ul of protein G agarose beads and incubated at 4°C with gentle shaking overnight. The samples were washed three times with 1xIP buffer (Sigma-Aldrich), combined with 50 μl of 1xSDS loading buffer, and separated by SDS-PAGE gel for immunoblot analysis using anti-GFP and anti-Myc antibody.

### *In planta* protein degradation assay and MG132 treatment

The plasmid *SPL11*:*Myc*, or *SPL11m*:*Myc*(m) was co-expressed with *GFP*:*SPIN6 plasmid* by agroinfiltration in *N*. *benthamiana*. The *RFP* plasmid was co-infiltrated as a control. Tissues were harvested 3 days after infiltration for protein extraction. For protein level analysis, total proteins were detected using anti-GFP, anti-Myc, and anti-RFP antibody. For MG132 treatment, 50 μM MG132 was infiltrated with DMSO at 18 h before tissue was sampled.

### *In vitro* GAP activity assay

The *Spin6* GAP domain fragment was cloned into the GST fusion vector pGEX-6p-1 (GE Healthcare). *OsRac1* cDNA was cloned into the MBP vector pMAL-c2 (New England BioLabs). The proteins were expressed and purified according to the manufacturer’s protocols for GAP activity assay. OsRac1 protein at 1 μM was loaded with 10 μM mant-GTP (Molecular Probes, M12415) in the following buffer: 20 mM Tris/HCl pH 8.0, 50 mM NaCl, and 1 mM EDTA. After 10 min at 25°C, the Mant-GTP loaded OsRac1 was used for the GAP activity assay by adding 5 μM SPIN6GAP or GST (control) proteins in reaction buffer (200 mM Tris-Cl pH 8.0, 500 mM NaCl, 10 mM EDTA). Samples were immediately placed into a Glo-MAX luminometer (Promega) for fluorescence detection (at 10 min intervals for 30 min) at 25°C. Each reaction was repeated three times, and means and standard errors are presented.

### Measurement of *in vivo* GAP activity of SPIN6

The Raichu intramolecular FRET sensor was used to analyze *in vivo* GAP activity of SPIN6 toward OsRac1 [21]. Ten to 12 hours after transfection of Raichu-OsRac1 into rice protoplasts, the cells were imaged using an Olympus IX-81 inverted microscope with a Yokogawa CSU22 confocal scanner equipped with the cooled charge-coupled device camera EM-CCD C9100–02 (Hamamatsu Photonics). Raichu-OsRac1 was excited using a 440-nm diode laser (iFLEX 2000, Point Source). The CFP and YFP filters were 480 ± 15 nm and 535 ± 20 nm, respectively. Back- ground fluorescence was subtracted, fluorescence bleed- through was normalized, and FRET efficiency was calculated according to published procedures [35].

### Sequence searching, alignment, and phylogenetic analysis

Sequences were collected using the BLAST program from the rice TIGR database (http://rice.plantbiology.msu.edu/) and the Arabidopsis TAIR database (http://www.arabidopsis.org/). For protein domain analysis, the SMART program was used (http://smart.embl-heidelberg.de/). The ClustalW 2.0 program [36] was used for sequence alignment, and GeneDoc (http://www.nrbsc.org/gfx/genedoc/) was used for conserved residue shading. For phylogenetic analysis, MEGA 3.1 [37] was used for building the neighbor-joining tree based on the bootstrap test (replications = 1000, random seeds = 24054).

## Supporting Information

S1 Text Supporting information figures. **Figure S1.** The cDNA alignment of *Spin6* and its two rice homologous genes. **Figure S2.** Protein sequence alignment of Pleckstrin Homology (PH)-Rho GTPase-activating proteins (RhoGAPs) in rice and Arabidopsis. The marked regions with underlines are PH, GAP and Coiled-Coil domains, respectively. **Figure S3.** Protein sequence alignment of the three SPIN6 splicing isoforms. **Figure S4.** Phylogenic analysis of RhoGAP proteins in rice, Arabidopsis and other organisms. (A) Phylogenic relationship of RhoGAP proteins in rice and Arabidopsis. CRIB: Cdc42/Rac-interactive binding domain, PRLP: Prokar lipoprotein domain, RhoGAP: Rho GTPase-activating protein domain, PH: Pleckstrin Homology PH domain. (B) Phylogenic relationship of RhoGAP proteins in rice, Arabidopsis, Human and *Drosophila*. **Figure S5.** Interacting domain analysis of SPIN6 and SPL11 by yeast two hybrid. (A) The domains of SPIN6 and SPL11 were used for yeast two-hybrid. SPIN6FL: SPIN6 full length, PH: SPIN6 PH domain, GAP: SPIN6 GAP domain, NT: SPIN6 N-terminal, CT: SPIN6 C-terminal, SPL11FL: SPL11 full length, ARM: SPL11 ARM domain. (B) Interacting domain analysis of SPIN6 and SPL11 in yeast. **Figure S6.** Yeast two-hybrid assay of the interaction between SPL11 and SPIN6 homolog Os03g24180, and SPIN6 and SPL11 homology OsPUB12 (Os06g01304). A. The interaction between SPL11 and Os03g24180. The full-length cDNA of Os03g24180 was cloned in the AD vector. The Y2H assay was carried out as described in Materials and Methods. B. The interaction between SPIN6 and OsPUB12 (Os06g01304). The E3 ligase domain was mutated in Os06g01304 and cloned into the AD vector to avoid self-activation in yeast. The Y2H assay was carried out as described in Materials and Methods. **Figure S7.** The controls of BiFC assay of SPL11, SPIN6 and OsRac1. NeYFP and CeYFP are blank controls, NeYFP was fused with SPL11, SPL11m, ARM, OsRac1, respectively, and CeYFP was fused with SPIN6. (A). BiFC assay between NeYFP and SPIN6:CeYFP; (B) BiFC assay between NeYFP:ARM and CeYFP; (C) BiFC assay between NeYFP:SPL11m and CeYFP; (D) BiFC assay between NeYFP:SPL11 and CeYFP; (E) BiFC assay between NeYFP:OsRac1 and CeYFP. **Figure S8.** Confirmation of SPIN6 ubiquitination by SPL11 with GST pulldown and MG132 inhibition of SPL11 degradation *in vivo*. (A). Ubiquitination of SPIN6 by SPL11 was further confirmed by GST pulldown. After the E3 ligase assay reaction, the GST:SPIN6 beads were washed with the PBST solution and subjected to immunoblot either with anti-ubiquitin or anti-GST antibody. Asterisk denotes GST:SPIN6 detected by the anti-GST antibody. (B). The same samples in Fig. 2 were used for immunoblot to detect SPL11:Myc with longer exposure time. With about 15 min of exposure, a faint band of SPL11:Myc was detected (second lane in the first panel) and as the exposure time was increased, the stronger band of SPL11:Myc was observed (second lane in the third panel, arrow indicated). **Figure S9.** *Spin6* RNAi construct map. A. An unique 302-bp 3’-UTR sequence of *Spin6* cDNA with no similarity with other sequences in the rice genome was cloned into the pANDA vector by Gateway cloning method. The two reverse UTR sequence fragments are linked by a Gus linker intron (1.0 kb). The Spin6 RNAi fragment is under the control of the maize ubiquitin promoter (1.9 kb). NOSt is the transcription terminator. The NPTII gene is for bacterial selection. HPT gene is for rice transgenic selection. B. Location and sequence of the RNAi fragment. The bold, masked sequence is the fragment for making the RNAi construct. The sequence with red font is *Spin6’s* coding region. The region with black font is either 3’ or 5’ UTR. **Figure S10.** Whole genome similarity searches of the *Spin6* RNAi fragment at the NCBI rice genome database. A. Mega BLAST search with the *Spin6* RNAi fragment. B. BLASTN search with the *Spin6* RNAi fragment. **Figure S11.** Phenotypes of the *Spin6* RNAi silencing and T-DNA insertion mutants at seedling (A) and mature (B) stages. NPB: Nipponbare, wild type; 16–2, 22–2 are two *Spin6* RNAi lines; Hwayoung: wild type of the T-DNA insertion mutant; *spin6*: T-DNA insertion mutant. **Figure S12.** Identification and genotyping of *Spin6* T-DNA insertion mutant. A. The T-DNA insertion localization in the *spin6* mutant. B. The PCR genotyping of the *spin6* mutant. Hwayoung: Wild type; *spin6*: a homozygous line of the *spin6* mutant. C. The expression pattern of *Spin6* and *Spl11* in the *spin6* mutant. The data represent average data of three replicates, the bar was shown by standard deviation (SD). **Figure S13.** Cell death and disease resistance phenotypes, and ROS generation after flg22 and chitin treatments of *spin6*. A. Cell death phenotypes of *spin6*. Hwayoung is the wild type. B. Phenotypes of inoculation with blast isolate RO1–1. C,D. Spores number and fungal biomass of lesions infected by rice blast isolate RO1–1. Rice ubiquitin (UBQ) was used as the internal control. E. Lesion length of *spin6* mutant and wild type (Hwayoung, HY) inoculated with Xoo race RB6. F, G. The ROS accumulation dynamcs in *spin6* mutant and wild type(HY) treated with chitin (F) and flg22 (G).Data means the average of three or more than three replicates, Error bar is SD. * and ** represent significant level at P<0.05 and P<0.01,respectively. **Figure S14.** Endogenous H_2_O_2_ content detection in *Spin6* RNAi lines(A) and T-DNA insertion mutant (B). NPB and Hwayoung are the wild type for the RNAi line and mutants, respectively. The data are the means of three replications with standard error as error bar. The significant level was at **P<0.01, n = 3 with t- test. **Figure S15.** Expression pattern of the defense-related genes *PAL*(A), *PR1a*(B), *OsRac1* (C, D), *OsSGT1* (E, F), and *OsRAR1* (G, H) in wild type Nipponbare (NPB) and *Spin6* RNAi plants (22–2) after chitin and flg22 treatments. The ubiquitin (UBQ) gene was used as the internal control. Data represents the means of three replications with standard error as error bar. The significant level was at **P<0.01, n = 3 with t test. **Figure S16.** The expression pattern of *Spin6*- and defense-related genes in wild type Nipponbare (NPB) and *Spin6* RNAi plants after inoculation with blast isolate R01–1. The leaf tissue was harvested at 0 (treated with water), 24 and 48 h after inoculated with *M*. *oryzae* isolate R01–1. Relative expression level of *Spin6*, *Spl11*, *RbohB*, *OsRac1*, *PR1a*, *OsNAC4*, *PR5* and *PBZ1* is shown in A, B, C, D, E, F, G and H, respectively. The relative transcriptional level of each gene was determined by real-time quantitative PCR using ubiquitin (UBQ) as the internal control. Error bars represent SD (n = 3). Significance was determined at *P<0.05 and **P<0.01 with a *t*-test. **Figure S17.** The flowering time of wild type Nipponbare (NPB) and *Spin6* RNAi (22–2) plants under both short day (SD) (A) and long day (LD) (B) conditions, and expression pattern of flowering marker genes *OsGI* (C), *Hd1* (D) and *Hd3a* (E) in both NPB and *Spin6* RNAi plants. The ubiquitin (UBQ) gene was used as the internal control. The data of each lines is the average of 5 plants, error bar represents SD. * and ** represent the significant level at P<0.05 and P<0.001, respectively
(PDF)

S2 Text Supporting information tables. **Table S1.** The cDNA sequence identity of *Spin6* and its two rice homologous genes. **Table S2.** The protein sequence identity of SPIN6 and its two rice homologous genes. **Table S3.** Primers used in this study.
(PDF)
