# Correction: The RhoGAP SPIN6 Associates with SPL11 and OsRac1 and Negatively Regulates Programmed Cell Death and Innate Immunity in Rice

**DOI:** 10.1371/journal.ppat.1004807

**Published:** 2015-04-17

**Authors:** The PLOS Pathogens Staff

The following information is missing from the Acknowledgments section:

"We thank Drs. Gautam Shirsekar and Jiangbo Fan who generated the data shown in Figure 3C, 3D, and 3E."
