# Supplementary material for: The RhoGAP SPIN6 Associates with SPL11 and OsRac1 and Negatively Regulates Programmed Cell Death and Innate Immunity in Rice
Source: PLoS Pathog. 2015 Feb 6;11(2):e1004629. doi: 10.1371/journal.ppat.1004629 (PMC4450066; doi:10.1371/journal.ppat.1004629)
Supplement: S1 Text — Figure S1. The cDNA alignment of Spin6 and its two rice homologous genes. Figure S2. Protein sequence alignment of Pleckstrin Homology (PH)-Rho GTPase-activating proteins (RhoGAPs) in rice and Arabidopsis. The marked regions with underlines are PH, GAP and Coiled-Coil domains, respectively. Figure S3. Protein sequence alignment of the three SPIN6 splicing isoforms. Figure S4. Phylogenic analysis of RhoGAP proteins in rice, Arabidopsis and other organisms. (A) Phylogenic relationship of RhoGAP proteins in rice and Arabidopsis. CRIB: Cdc42/Rac-interactive binding domain, PRLP: Prokar lipoprotein domain, RhoGAP: Rho GTPase-activating protein domain, PH: Pleckstrin Homology PH domain. (B) Phylogenic relationship of RhoGAP proteins in rice, Arabidopsis, Human and Drosophila. Figure S5. Interacting domain analysis of SPIN6 and SPL11 by yeast two hybrid. (A) The domains of SPIN6 and SPL11 were used for yeast two-hybrid. SPIN6FL: SPIN6 full length, PH: SPIN6 PH domain, GAP: SPIN6 GAP domain, NT: SPIN6 N-terminal, CT: SPIN6 C-terminal, SPL11FL: SPL11 full length, ARM: SPL11 ARM domain. (B) Interacting domain analysis of SPIN6 and SPL11 in yeast. Figure S6. Yeast two-hybrid assay of the interaction between SPL11 and SPIN6 homolog Os03g24180, and SPIN6 and SPL11 homology OsPUB12 (Os06g01304). A. The interaction between SPL11 and Os03g24180. The full-length cDNA of Os03g24180 was cloned in the AD vector. The Y2H assay was carried out as described in Materials and Methods. B. The interaction between SPIN6 and OsPUB12 (Os06g01304). The E3 ligase domain was mutated in Os06g01304 and cloned into the AD vector to avoid self-activation in yeast. The Y2H assay was carried out as described in Materials and Methods. Figure S7. The controls of BiFC assay of SPL11, SPIN6 and OsRac1. NeYFP and CeYFP are blank controls, NeYFP was fused with SPL11, SPL11m, ARM, OsRac1, respectively, and CeYFP was fused with SPIN6. (A). BiFC assay between NeYFP and SPIN6:CeYFP; (B) BiFC assay between NeYFP:AR [file ppat.1004629.s001.pdf]

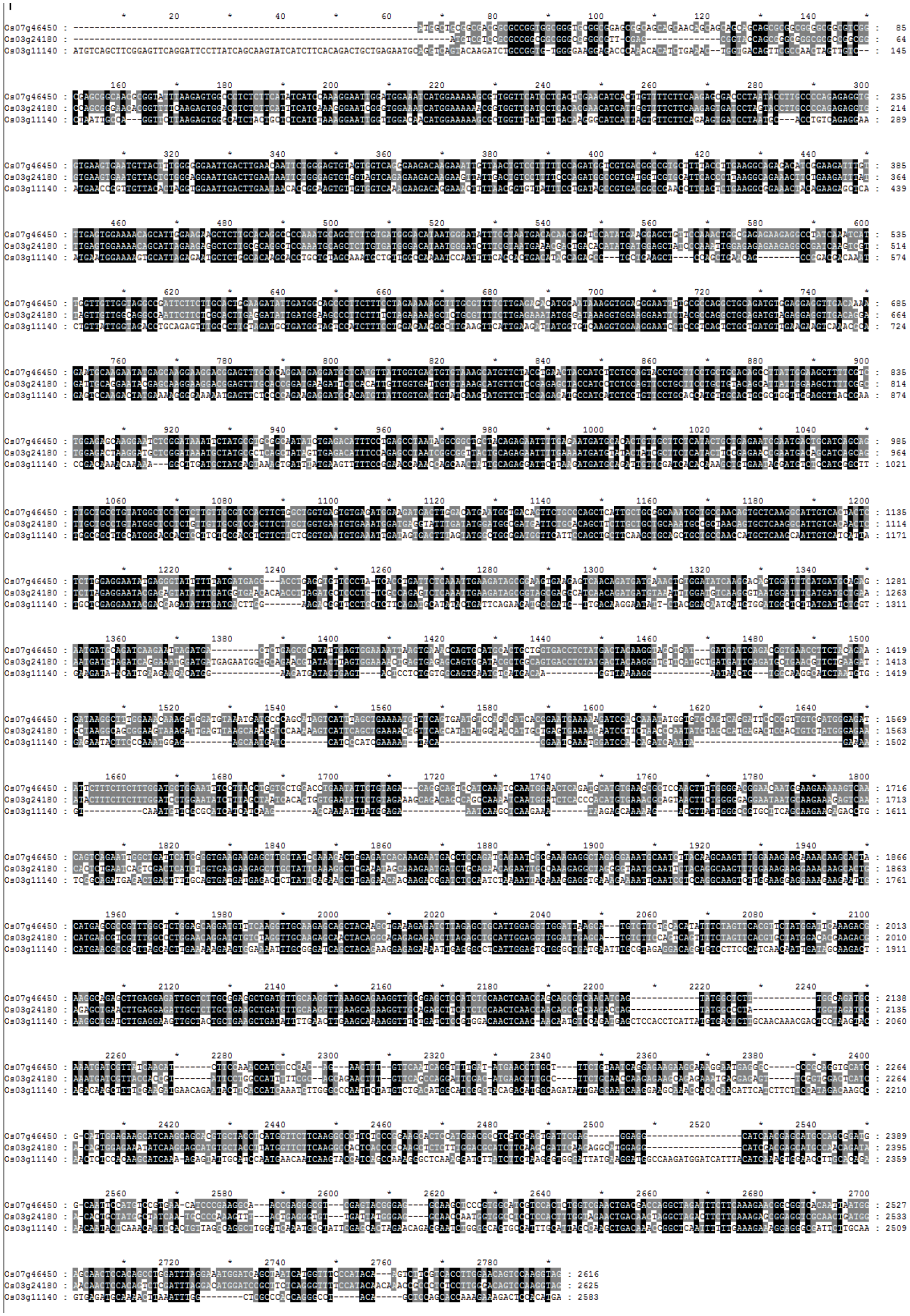

Figure S1. The cDNA alignment of *Spin6* and its two rice homologous genes.

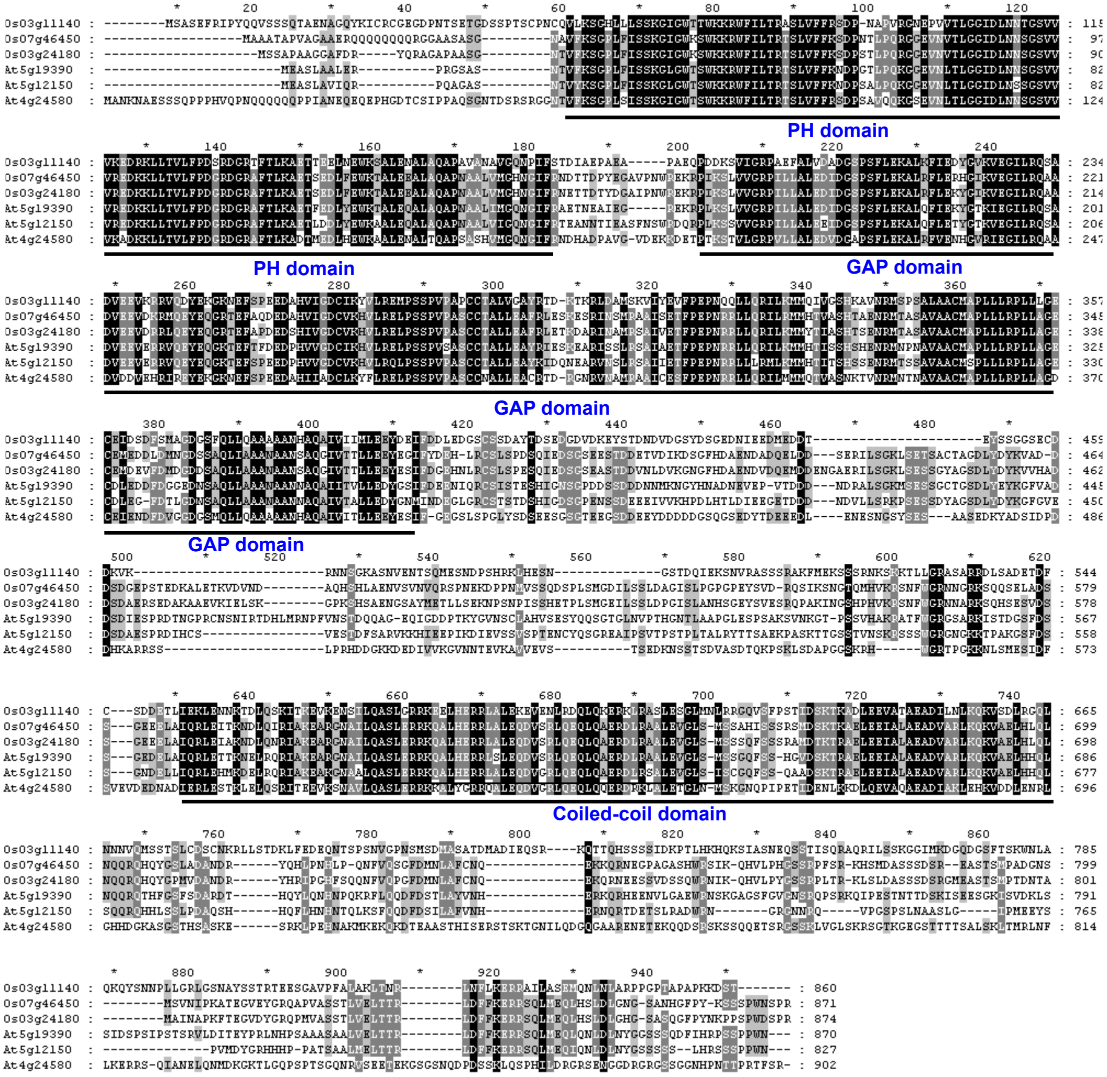

Figure S2. Protein sequence alignment of Pleckstrin Homology (PH)-Rho GTPase-activating proteins (RhoGAPs) in rice and Arabidopsis. The marked regions with underlines are PH , GAP and Coiled-Coil domains, respectively.

```
                  *        20         *        40         *        60         *        80         *       100
SPIN6.1 : MAAATAPVAGAAERQQQQQQQQRGGAASASGNAVFKSGPLFISSKGIGWKSWKKRWFILTRTSLVFFKSDPNTLPQRGGEVNVTLGGIDLNNSGSVVVRE : 100
SPIN6.2 : ---------------------------------------------------------------------------------------------------- :   -
SPIN6.3 : ---------------------------------------------------------------------------------------------------- :   -

                  *       120         *       140         *       160         *       180         *       200
SPIN6.1 : DKKLLTVLFPDGRDGRAFTLKAETSEDLFEWKTALEEALAQAPNAALVMGHNGIFRNDTTDPYEGAVPNWREKRPIKSLVVGRPILLALEDIDGSPSFLE : 200
SPIN6.2 : ---------------------------------------------------------------------------------------------------- :   -
SPIN6.3 : ---------------------------------------------------------------------------------------------------- :   -

                  *       220         *       240         *       260         *       280         *       300
SPIN6.1 : KALRFLERHGIKVEGILRQAADVEEVDKRMQEYEQGRTEFAQDEDAHVIGDCVKHVLRELPSSPVPASCCTALLEAFRLESKESRINSMRAAISETFPEP : 300
SPIN6.2 : ---------------------------------------------------------------------------------------------------- :   -
SPIN6.3 : ----------------------------------------------------------------------------------------MRAAISETFPEP :  12

                  *       320         *       340         *       360         *       380         *       400
SPIN6.1 : NRRLLQRILRMMHTVASHTAENRMTASAVAACMAPLLLRPLLAGECEMEDDLDMNGDSSAQLIAAANAANSAQGIVTTLLEEYEGIFYDEHLRCSLSPDS : 400
SPIN6.2 : ---------------------------------------------------------------------------------------------------- :   -
SPIN6.3 : NRRLLQRILRMMHTVASHTAENRMTASAVAACMAPLLLRPLLAGECEMEDDLDMNGDSSAQLIAAANAANSAQGIVTTLLEEYEGIFYDEHLRCSLSPDS : 112

                  *       420         *       440         *       460         *       480         *       500
SPIN6.1 : QIEDSGSEESTDDETVDIKDSGFHDAENDADQELDDSERILSGKLSETSACTAGDLYDYKVADDDSDGEPSTEDKALETKVDVNDAQHSHLAENVSVNVQ : 500
SPIN6.2 : ---------------------------------------------------------------------------------------------------- :   -
SPIN6.3 : QIEDSGSEESTDDETVDIKDSGFHDAENDADQELDDSERILSGKLSETSACTAGDLYDYKVADDDSDGEPSTEDKALETKVDVNDAQHSHLAENVSVNVQ : 212

                  *       520         *       540         *       560         *       580         *       600
SPIN6.1 : RSPNEKDPPNMVSSQDSPLSMGDILSSLDAGISLPGPGPEYSVDRQSIKSNGTQMHVKRSNFWGRNNGRKSQQSELADSSGEEELAIQRLEITKNDLQIR : 600
SPIN6.2 : ----------MVSSQDSPLSMGDILSSLDAGISLPGPGPEYSVDRQSIKSNGTQMHVKRSNFWGRNNGRKSQQSELADSSGEEELAIQRLEITKNDLQIR :  90
SPIN6.3 : RSPNEKDPPNMVSSQDSPLSMGDILSSLDAGISLPGPGPEYSVDRQSIKSNGTQMHVKRSNFWGRNNGRKSQQSELADSSGEEELAIQRLEITKNDLQIR : 312

                  *       620         *       640         *       660         *       680         *       700
SPIN6.1 : IAKEARGNAILQASLERRKQALHERRLALEQDVSRLQEQLQAERDLRAALEVGLSMSSAHISSSRSMDSKTKAELEEIALAEADVARLKQKVAELHLQLN : 700
SPIN6.2 : IAKEARGNAILQASLERRKQALHERRLALEQDVSRLQEQLQAERDLRAALEVGLSMSSAHISSSRSMDSKTKAELEEIALAEADVARLKQKVAELHLQLN : 190
SPIN6.3 : IAKEARGNAILQASLERRKQALHERRLALEQDVSRLQEQLQAERDLRAALEVGLSMSSAHISSSRSMDSKTKAELEEIALAEADVARLKQKVAELHLQLN : 412

                  *       720         *       740         *       760         *       780         *       800
SPIN6.1 : QQRQHQYGSLADANDRYQHLPNHLPQNFVQSGFDMNLAFCNQEKKQRNE-GPAGASHWRSIKQHVLPHGSSRPFSRKHSMDASSSDSREASTSMPADGNS : 799
SPIN6.2 : QQRQHQYGSLADANDRYQHLPNHLPQNFVQSGFDMNLAFCNQEKKQRNEQGPAGASHWRSIKQHVLPHGSSRPFSRKHSMDASSSDSREASTSMPADGNS : 290
SPIN6.3 : QQRQHQYGSLADANDRYQHLPNHLPQNFVQSGFDMNLAFCNQEKKQRNE-----VS-------------------------------------------- : 463

                  *       820         *       840         *       860         *
SPIN6.1 : MSVNIPKATEGVEYGRQAPVASSTLVELTTRLDFFKERRSQLMEQLHSLDLGNGSANHGFPYKSSSPWNSPR : 871
SPIN6.2 : MSVNIPKATEGVEYGRQAPVASSTLVELTTRLDFFKERRSQLMEQLHSLDLGNGSANHGFPYKSSSPWNSPR : 362
SPIN6.3 : ------------------------------------------------------------------------ :   -
```

Figure S3. Protein sequence alignment of the three SPIN6 splicing isoforms.

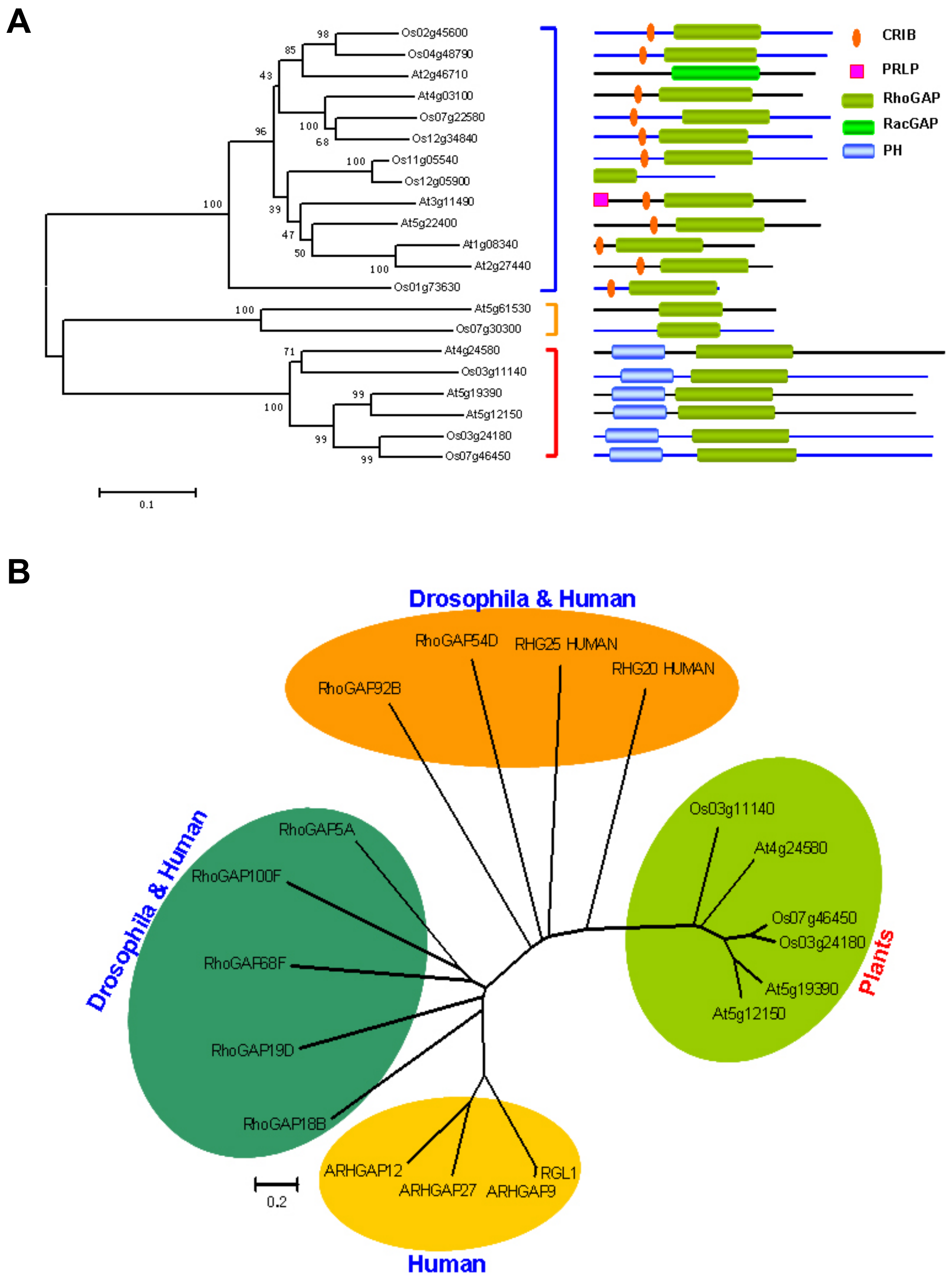

Figure S4. Phylogenic analysis of RhoGAP proteins in rice, Arabidopsis and other organisms. (**A**) Phylogenic relationship of RhoGAP proteins in rice and Arabidopsis. CRIB: Cdc42/Rac-interactive binding domain, PRLP: Prokar lipoprotein domain, RhoGAP: Rho GTPase-activating protein domain, PH: Pleckstrin Homology PH domain. (**B**) Phylogenic relationship of RhoGAP proteins in rice, Arabidopsis, Human and Drosophila.

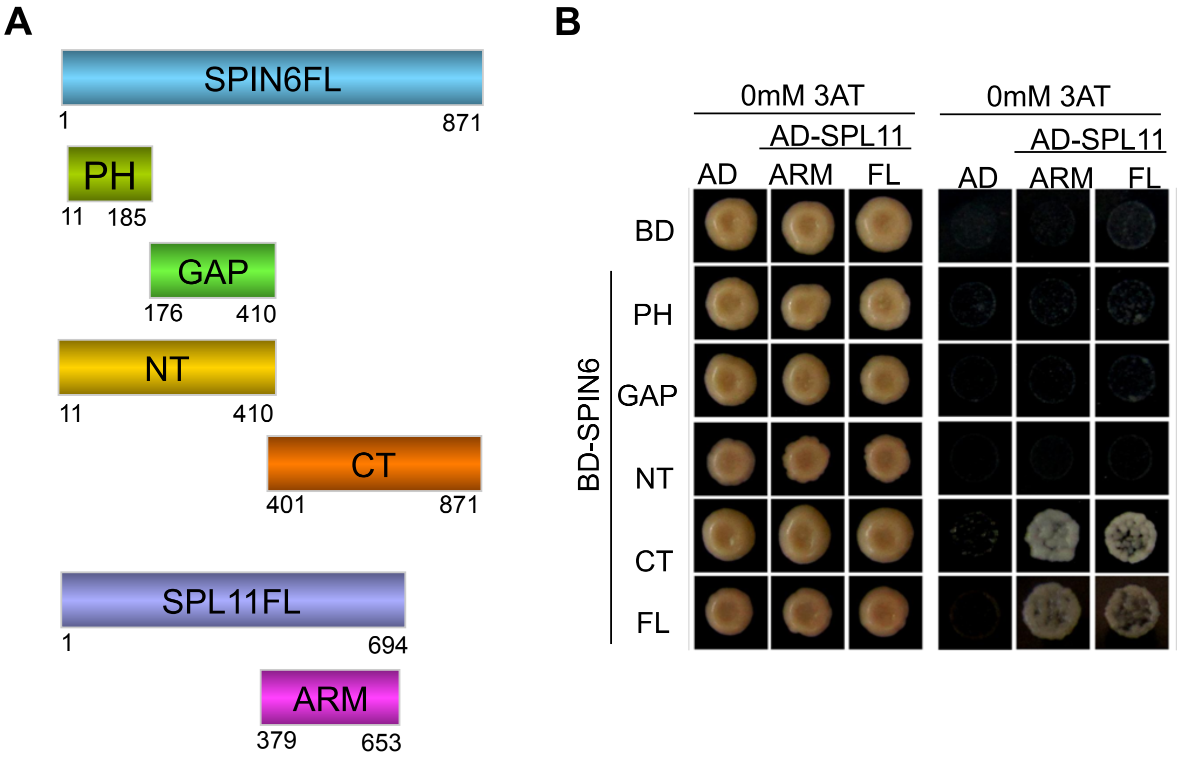

Figure S5. Interacting domain analysis of SPIN6 and SPL11 by yeast two hybrid. (**A**) The domains of SPIN6 and SPL11 were used for yeast two-hybrid. SPIN6FL: SPIN6 full length, PH: SPIN6 PH domain, GAP: SPIN6 GAP domain, NT: SPIN6 N-terminal, CT: SPIN6 C-terminal, SPL11FL: SPL11 full length, ARM: SPL11 ARM domain. (**B**) Interacting domain analysis of SPIN6 and SPL11 in yeast.

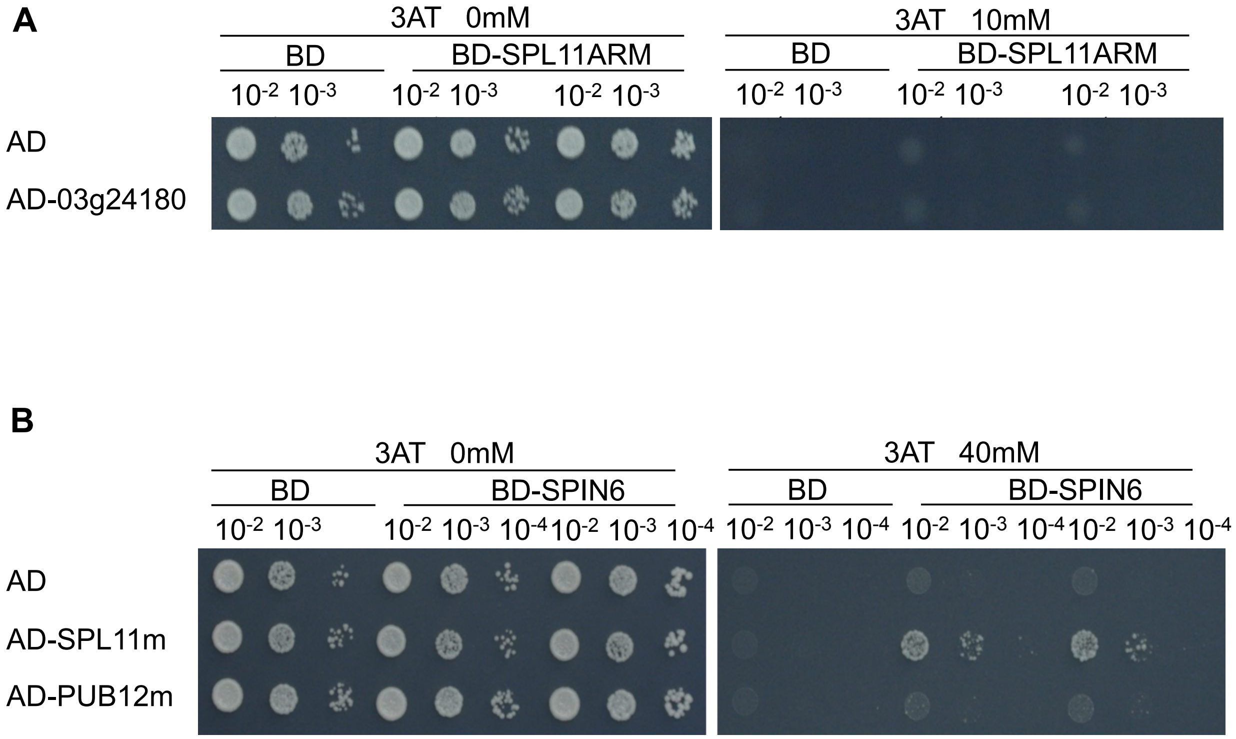

Figure S6. Yeast two-hybrid assay of the interaction between SPL11 and SPIN6 homolog Os03g24180, and SPIN6 and SPL11 homology OsPUB12 (Os06g01304). A. The interaction between SPL11 and Os03g24180. The full-length cDNA of Os03g24180 was cloned in the AD vector. The Y2H assay was carried out as described in Materials and Methods. B. The interaction between SPIN6 and OsPUB12 (Os06g01304). The E3 ligase domain was mutated in Os06g01304 and cloned into the AD vector to avoid self-activation in yeast. The Y2H assay was carried out as described in Materials and Methods.

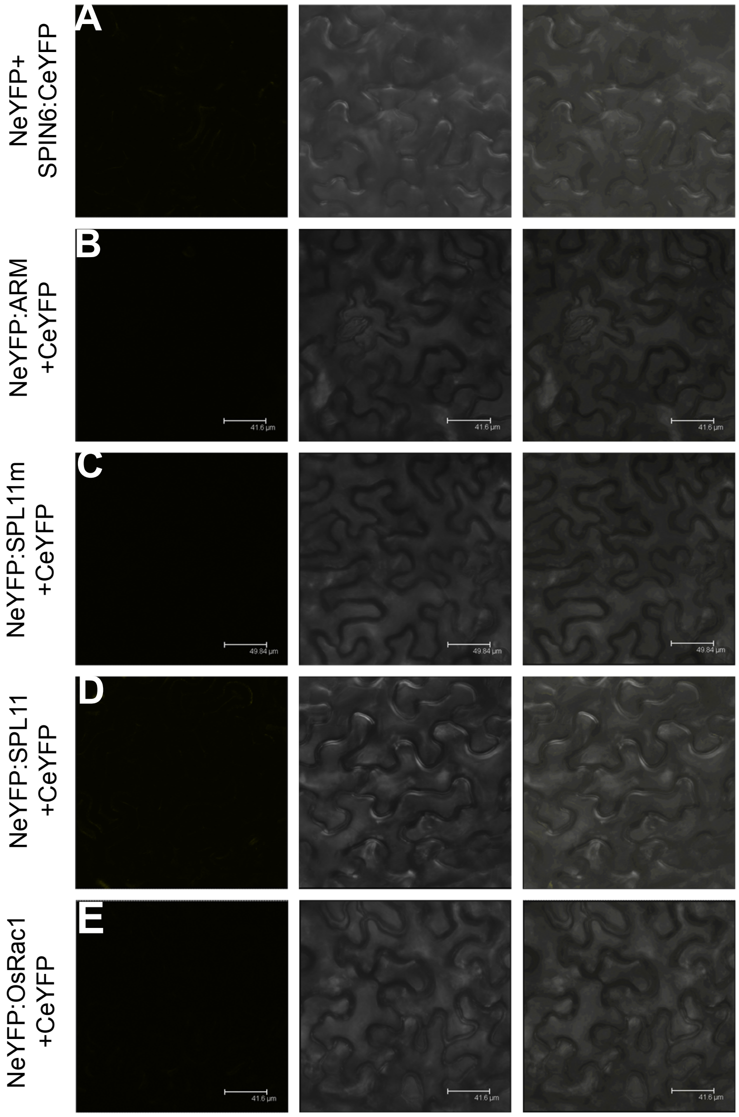

Figure S7. The controls of BiFC assay of SPL11, SPIN6 and OsRac1. NeYFP and CeYFP are blank controls, NeYFP was fused with SPL11, SPL11m, ARM, OsRac1, respectively, and CeYFP was fused with SPIN6. (A). BiFC assay between NeYFP and SPIN6:CeYFP; (B) BiFC assay between NeYFP:ARM and CeYFP; (C) BiFC assay between NeYFP:SPL11m and CeYFP; (D) BiFC assay between NeYFP:SPL11 and CeYFP; (E) BiFC assay between NeYFP:OsRac1 and CeYFP.

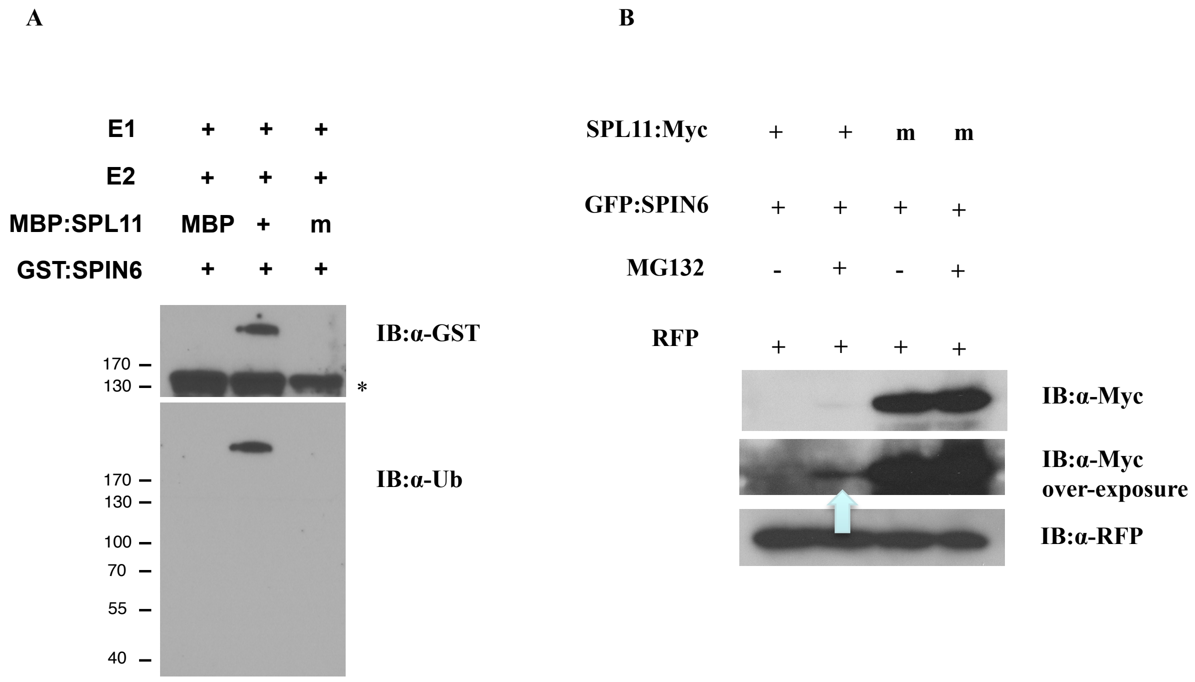

Figure S8. Confirmation of SPIN6 ubiquitination by SPL11 with GST pulldown and MG132 inhibition of SPL11 degradation *in vivo.* (A). Ubiquitination of SPIN6 by SPL11 was further confirmed by GST pulldown. After the E3 ligase assay reaction, the GST:SPIN6 beads were washed with the PBST solution and subjected to immunoblot either with anti-ubiquitin or anti-GST antibody. Asterisk denotes GST:SPIN6 detected by the anti-GST antibody. (B). The same samples in Figure 2 were used for immunoblot to detect SPL11:Myc with longer exposure time. With about 15 min of exposure, a faint band of SPL11:Myc was detected (second lane in the first panel) and as the exposure time was increased, the stronger band of SPL11:Myc was observed (second lane in the third panel, arrow indicated).

A

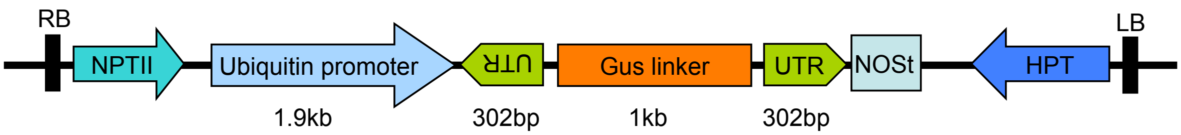

B

>Spin6 cDNA sequence

5′-GACGCGCCCACGCCCACCACCACCACCACCTCCATGACTGCATCCTCCTCCAAAAACCCAAGGCACAAATCCTAACCACCGGACGCGAG
CCGACGCGACGCGACGCGAGTCCCCATCCACATCGCTCGCGGCCCTTCCACCGCACGCCAGATCGGTGGCGGCGGCTGCTCGGGGTTG
GATCCAATGCGAGCGGTGATTGATAGCTGTTGATGCGAAGGACGACGATCTGAGGTGGTGGTGGAGTTGGGGGGATGGCTGCGGCGACGGC
GCCGGTGGCGGGTGCGGCGGAGCGGCAGCAGCAACAGCAGCAGCAGCAGCGCGGCGGGGCGGCGTCGGCGAGCGGCAACGCGGTATTTAAG
AGTGGCCCTCTCTTCATATCATCCAAAGGAATTGGATGGAAATCATGGAAAAAGCGTTGGTTCATCCTCACTCGAACATCACTTGTTTTCT
TCAAGAGCGACCCTAATACCTTGCCCCAGAGAGGTGGTGAAGTGAATGTTACTTTGGGGGGAATTGACTTGAACAATTCTGGGAGTGTAGT
GGTCAGGGAAGACAAGAAATTGTTAACTGTCCTTTTTCCAGATGGTCGTGACGGCCGTGCTTTTACCTTGAAGGCAGAGACATCGGAAGAT
TTGTTTGAGTGGAAAACAGCATTGGAAGAAGCTCTTGCACAGGCCCCAAATGCAGCTCTTGTGATGGGACATAATGGGATATTTCGTAATG
ACACAACAGATCCATATGAAGGAGCTGTTCCAAACTGGCGAGAGAAGAGGCCTATCAAATCATTGGTTGTTGGTAGGCCGATTCTTCTTGC
ACTGGAAGATATTGATGGCAGCCCTTCTTTCCTAGAAAAAGCTTTGCGTTTTCTTGAGAGACATGGAATAAAGGTGGAGGGAATTTTGCGC
CAGGCTGCAGATGTGGAGGAGGTTGACAAAAGAATGCAAGAATATGAGCAAGGAAGGACGGAGTTTGCACAGGATGAGGATGCTCATGTTA
TTGGTGACTGTGTAAAGCATGTTCTACGTGAACTACCATCTTCTCCAGTACCTGCTTCCTGCTGCACAGCCTTATTGGAAGCTTTTCGTCT
GGAGAGCAAGGAATCTCGGATAAATTCTATGCGTGCGGCAATATCTGAGACATTTCCTGAGCCTAATAGGCGGCTGCTACAGAGAATTTTG
AGAATGATGCACACTGTTGCTTCTCATACTGCTGAGAATCGAATGACTGCATCAGCAGTTGCTGCCTGTATGGCTCCTCTCTTGTTGCGTC
CACTTCTGGCTGGTGAGTGTGAGATGGAAGATGACTTGGACATGAATGGTGACAGTTCTGCCCAGCTCATTGCTGCGGCAAATGCTGCCAA
CAGTGCTCAAGGCATTGTCACTACTCTCTTGGAGGAATATGAGGGTATTTTTTATGATGAGCACCTGAGGTGTTCCCTATCACCTGATTCT
CAAATTGAAGATAGCGGAAGTGAAGAGTCAACAGATGATGAAACTGTGGATATCAAGGACAGTGGATTTCATGATGCAGAGAATGATGCAG
ATCAAGAATTAGATGACTCTGAGCGCATATTGAGTGGAAAATTAAGTGAAACCAGTGCATGCACTGCTGGTGACCTCTATGACTACAAGGT
AGCTGATGATGATTCAGACGGTGAACCTTCTACAGAAGATAAGGCTTTGGAAACAAAGGTGGATGTAAATGATGCCCAGCATAGTCATTTA
GCTGAAAATGTTTCAGTGAATGTCCAGAGATCACCGAATGAAAAAGATCCACCAAATATGGTGTCCAGTCAGGATTCCCCGTTGTCGATGG
GAGATATTCTTTCTTCTTTGGATGCTGGAATTTCCTTACCTGGTCCTGGACCTGAATATTCTGTAGACAGGCAGTCCATCAAATCCAATGG
AACTCAGATGCATGTGAAGCGCTCCAACTTTTGGGGACGGAACAATGGAAGAAAAAGTCAACAGTCAGAATTGGCTGATTCATCGGGTGAA
GAAGAGCTTGCTATCCAAAGACTGGAGATCACAAAGAATGACCTCCAGATCAGAATCGCGAAAGAGGCTAGAGGAAATGCAATCTTACAAG
CAAGTTTGGAAAGAAGAAAACAAGCACTACATGAGCGCCGTTTGGCTCTGGAGCAGGATGTTTCAAGGTTGCAAGAGCAGCTACAAGCTGA
AAGAGATCTTAGAGCTGCATTGGAGGTTGGATTAAGCATGTCTTCTGCACATATTTCTAGTTCACGTTCTATGGATTCAAAGACGAAGGCA
GAGCTTGAGGAGATTGCTCTTGCGGAGGCTGATGTTGCAAGGTTAAAGCAGAAGGTTGCGGAGCTCCATCTCCAACTCAACCAGCAGCGTC
AACATCAGTATGGCTCTTTGGCAGATGCAAATGATCGTTATCAACATCTTCCAAACCATCTCCCACAGAACTTTGTTCAATCAGGTTTTGA
TATGAACCTTGCTTTCTGTAATCAGGAGAAGAAGCAAAGGAATGAGGGCCCCGCAGGTGCATCGCATTGGAGAAGCATCAAGCAGCACGTG
CTACCTCATGGTTCTTCAAGGCCCTTCTCCCGGAAGCACTCCATGGACGCCTCGTCGAGTGATTCGAGGGAGGCATCAACGAGCATGCCAG
CGGATGGCAATTCCATGTCCGTGAACATCCCGAAGGCAACCGAGGGCGTCGAGTACGGGAGGCAAGCTCCCGTGGCATCGTCCACTCTGGT
CGAACTGACGACCAGGCTAGATTTCTTCAAAGAACGGCGGT**CACAATTAATGGAGCAACTCCACAGCCTGGATTTAGGAAATGGATCAGCT**
AATCATGGTTTCCCATACAAGTCTTCGTCACCTTGGAACAGTCCAAGGTAGGAGGAGACTTCCTTTTTTTTGCGACATTCTGTTTGTATAT
TCTTGGTAAAGAATCCTCTCGTTTTCTTTCGTAGAAGCTGCTAGTGAGAATGAATGTATATCCCTTTCCTGTTCGTCTTCCATTTGTTTTT
GTTTTGTTGTTGCATGCGGCAGCCTCTTTTGGGTGTGATTTTGTAATATGCTTTTTGTAATCTATTGTATGTAACACGAGTTGGGGGATC
ATAATCATTGCCACTTGTTTCTGGTGTCTTTCC-3′

Figure S9. *Spin6* RNAi construct map. **A.** An unique 302-bp 3’-UTR sequence of *Spin6* cDNA with no similarity with other sequences in the rice genome was cloned into the pANDA vector by Gateway cloning method. The two reverse UTR sequence fragments are linked by a Gus linker intron (1.0 kb). The Spin6 RNAi fragment is under the control of the maize ubiquitin promoter (1.9 kb). NOSt is the transcription terminator. The NPTII gene is for bacterial selection. HPT gene is for rice transgenic selection. **B.** Location and sequence of the RNAi fragment. The bold, masked sequence is the fragment for making the RNAi construct. The sequence with red font is *Spin6’s* coding region. The region with black font is either 3’ or 5’ UTR.

A

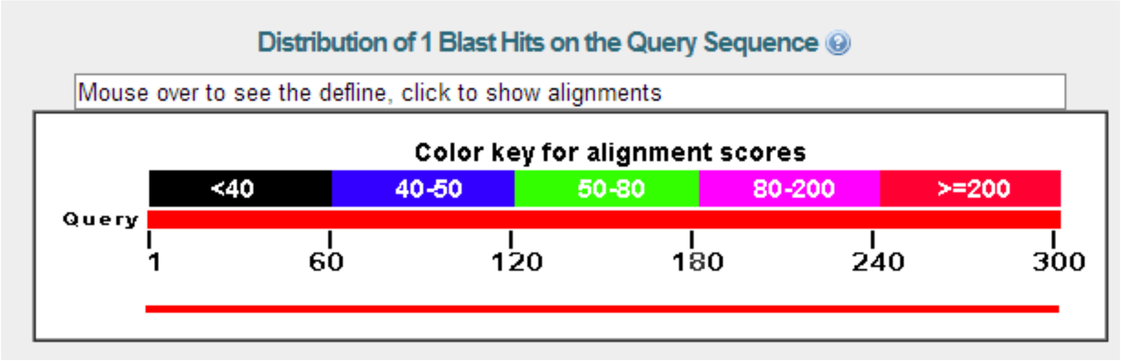

B

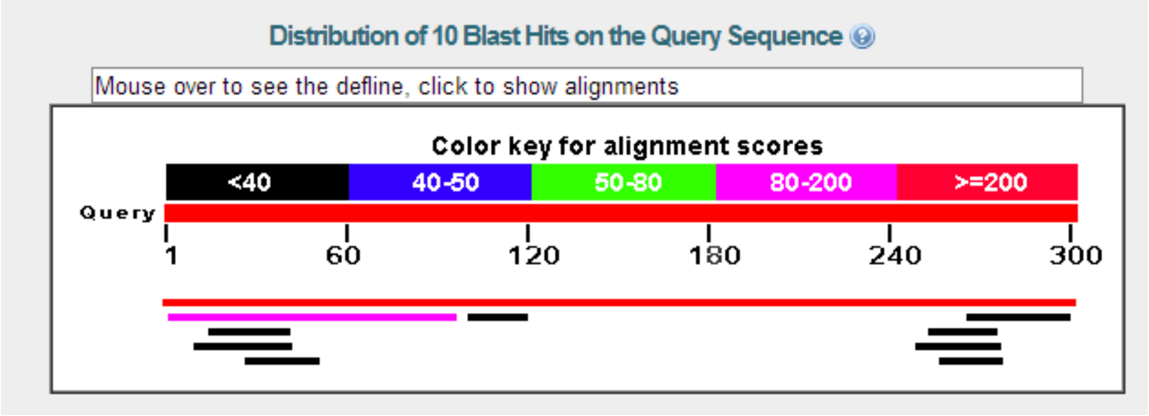

Figure S10. Whole genome similarity searches of the *Spin6* RNAi fragment at the NCBI rice genome database. **A.** Mega BLAST search with the *Spin6* RNAi fragment. **B.** BLASTN search with the *Spin6* RNAi fragment.

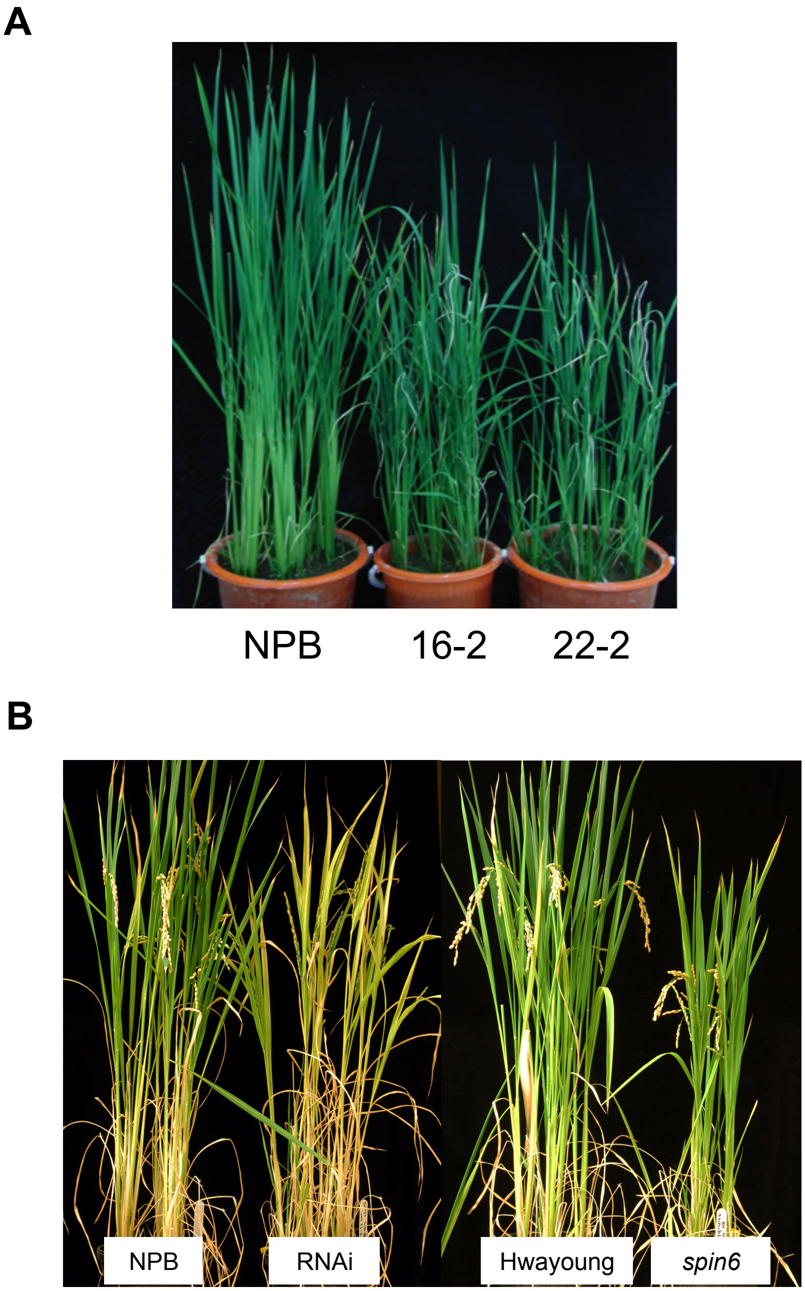

Figure S11. Phenotypes of the *Spin6* RNAi silencing and T-DNA insertion mutants at seedling (A) and mature (B) stages. NPB: Nipponbare, wild type; 16-2, 22-2 are two *Spin6* RNAi lines; Hwayoung: wild type of the T-DNA insertion mutant; *spin6*: T-DNA insertion mutant.

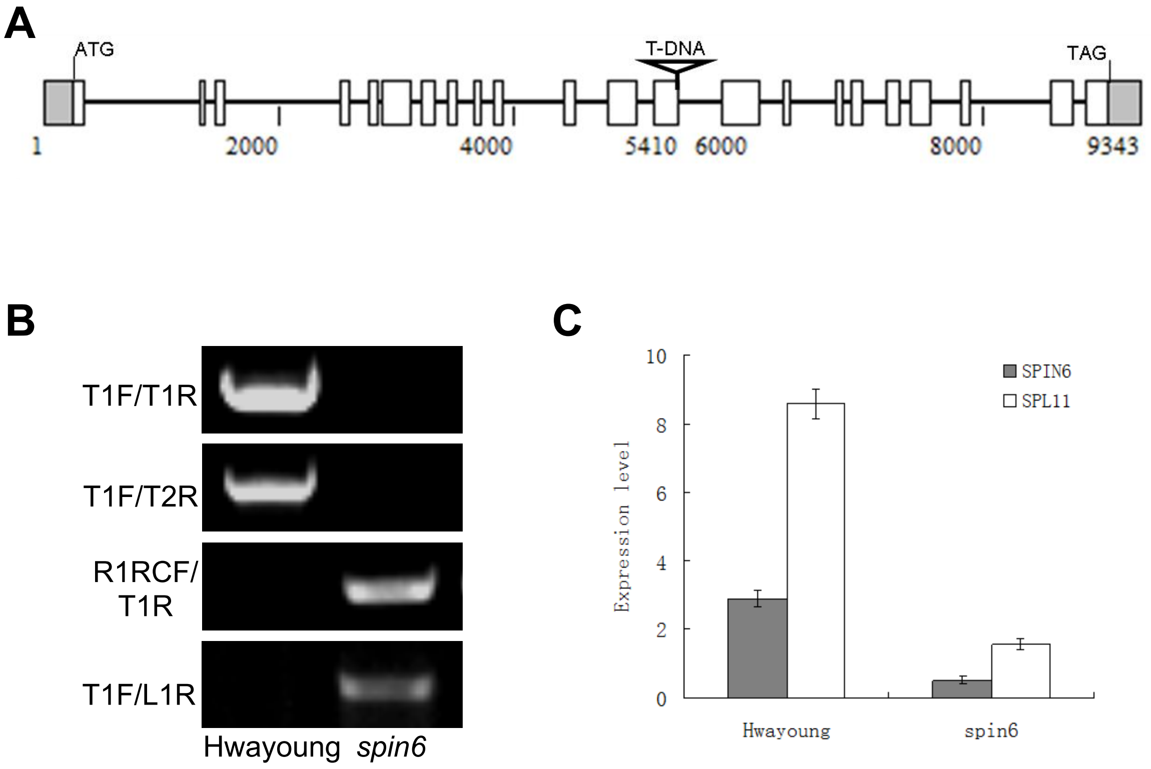

Figure S12. Identification and genotyping of *Spin6* T-DNA insertion mutant. **A.** The T-DNA insertion localization in the *spin6* mutant. **B.** The PCR genotyping of the *spin6* mutant. Hwayoung: Wild type; *spin6*: a homozygous line of the *spin6* mutant. **C**. The expression pattern of *Spin6* and *Spl11* in the *spin6* mutant. The data represent average data of three replicates, the bar was shown by standard deviation (SD).

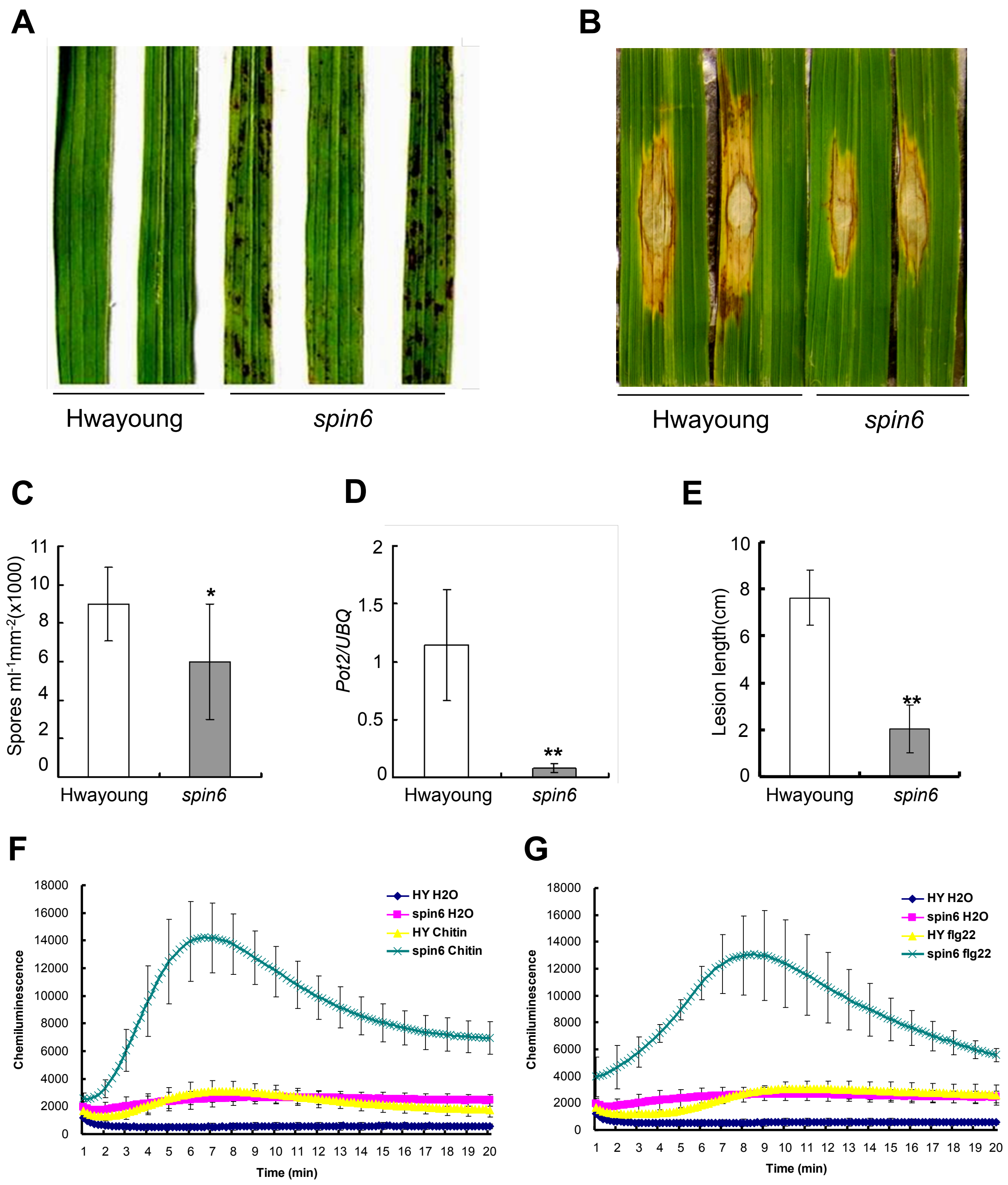

Figure S13. Cell death and disease resistance phenotypes, and ROS generation after flg22 and chitin treatments of *spin6*. A. Cell death phenotypes of *spin6*. Hwayoung is the wild type. B. Phenotypes of inoculation with blast isolate RO1-1. C,D. Spores number and fungal biomass of lesions infected by rice blast isolate RO1-1. Rice ubiquitin (UBQ) was used as the internal control. E. Lesion length of *spin6* mutant and wild type (Hwayoung, HY) inoculated with Xoo race RB6. F, G. The ROS accumulation dynamcs in *spin6* mutant and wild type(HY) treated with chitin (F) and flg22 (G).Data means the average of three or more than three replicates, Error bar is SD. * and ** represent significant level at $P<0.05$ and $P<0.01$,respectively.

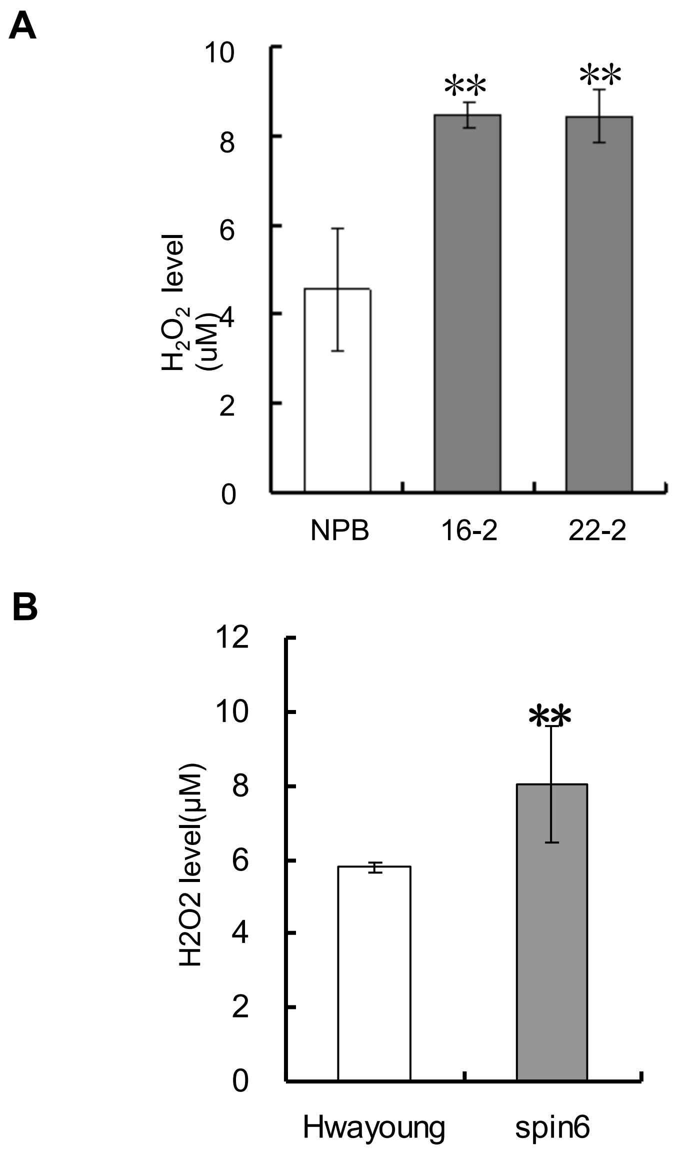

Figure S14. Endogenous H_2O_2 content detection in *Spin6* RNAi lines(A) and T-DNA insertion mutant (B). NPB and Hwayoung are the wild type for the RNAi line and mutants, respectively. The data are the means of three replications with standard error as error bar. The significant level was at **P<0.01, n=3 with t- test.

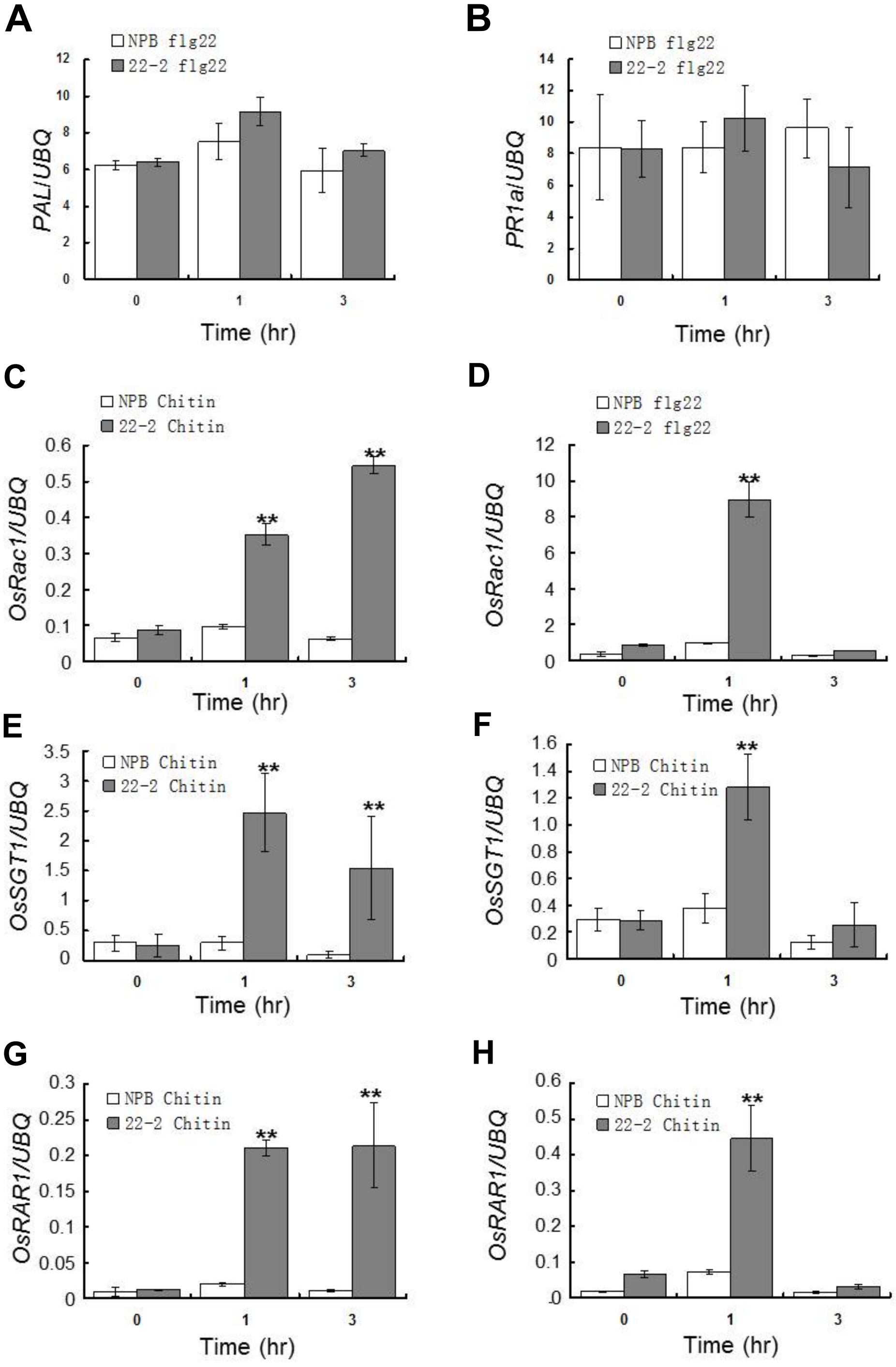

Figure S15. Expression pattern of the defense-related genes *PAL*(A), *PR1a*(B), *OsRac1* (C, D), *OsSGT1* (E, F), and *OsRAR1* (G, H) in wild type Nipponbare (NPB) and *Spin6* RNAi plants (22-2) after chitin and flg22 treatments. The ubiquitin (UBQ) gene was used as the internal control. Data represents the means of three replications with standard error as error bar. The significant level was at **P<0.01, n=3 with t test.

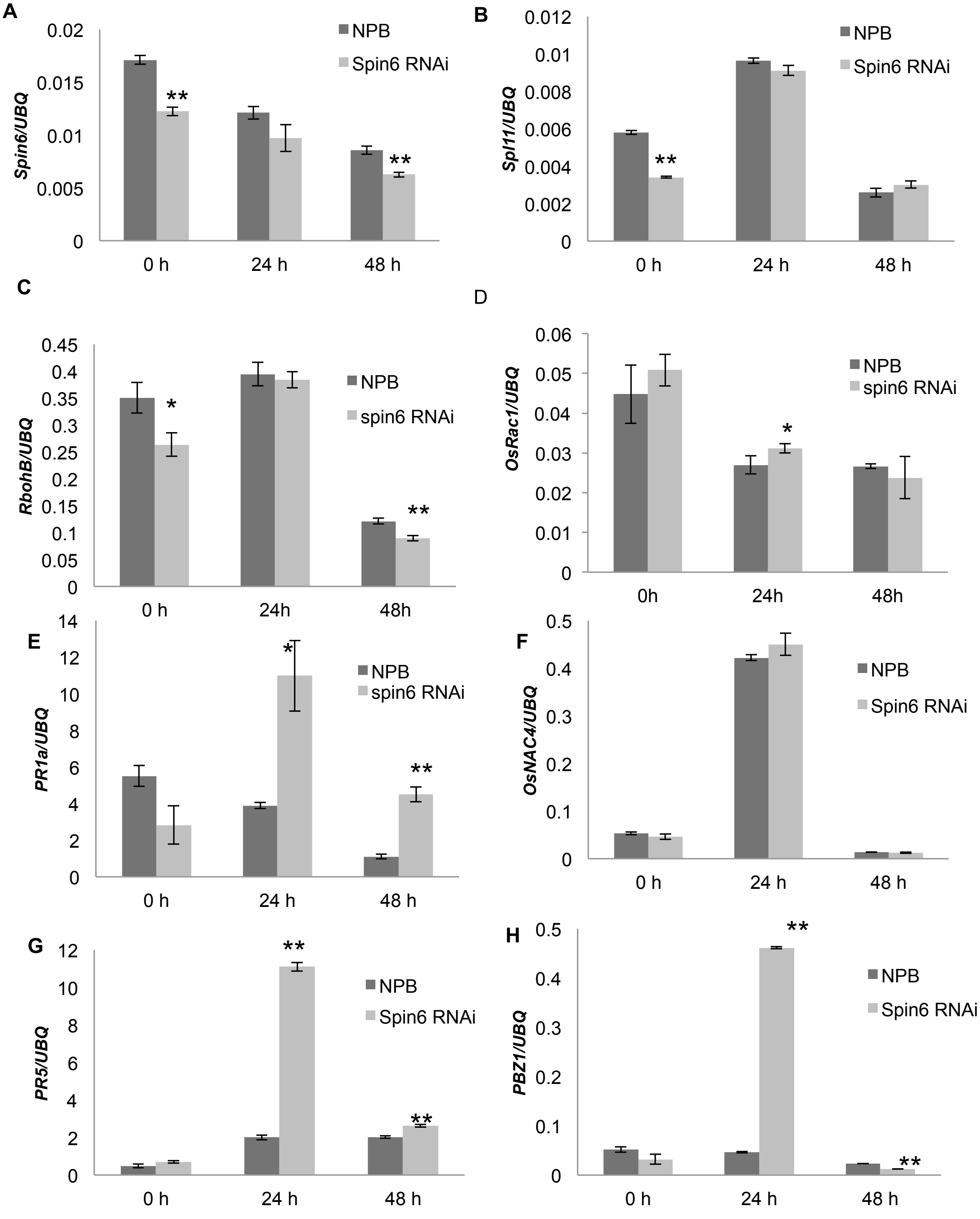

Figure S16. The expression pattern of *Spin6*- and defense-related genes in wild type Nipponbare (NPB) and *Spin6* RNAi plants after inoculation with blast isolate R01-1. The leaf tissue was harvested at 0 (treated with water), 24 and 48 h after inoculated with *M. oryzae* isolate R01-1. Relative expression level of *Spin6, Spl11, RbohB, OsRac1, PR1a, OsNAC4, PR5* and *PBZ1* is shown in A, B, C, D, E, F, G and **H**, respectively. The relative transcriptional level of each gene was determined by real-time quantitative PCR using ubiquitin (UBQ) as the internal control. Error bars represent SD (n=3). Significance was determined at *P<0.05 and **P<0.01 with a *t*-test.

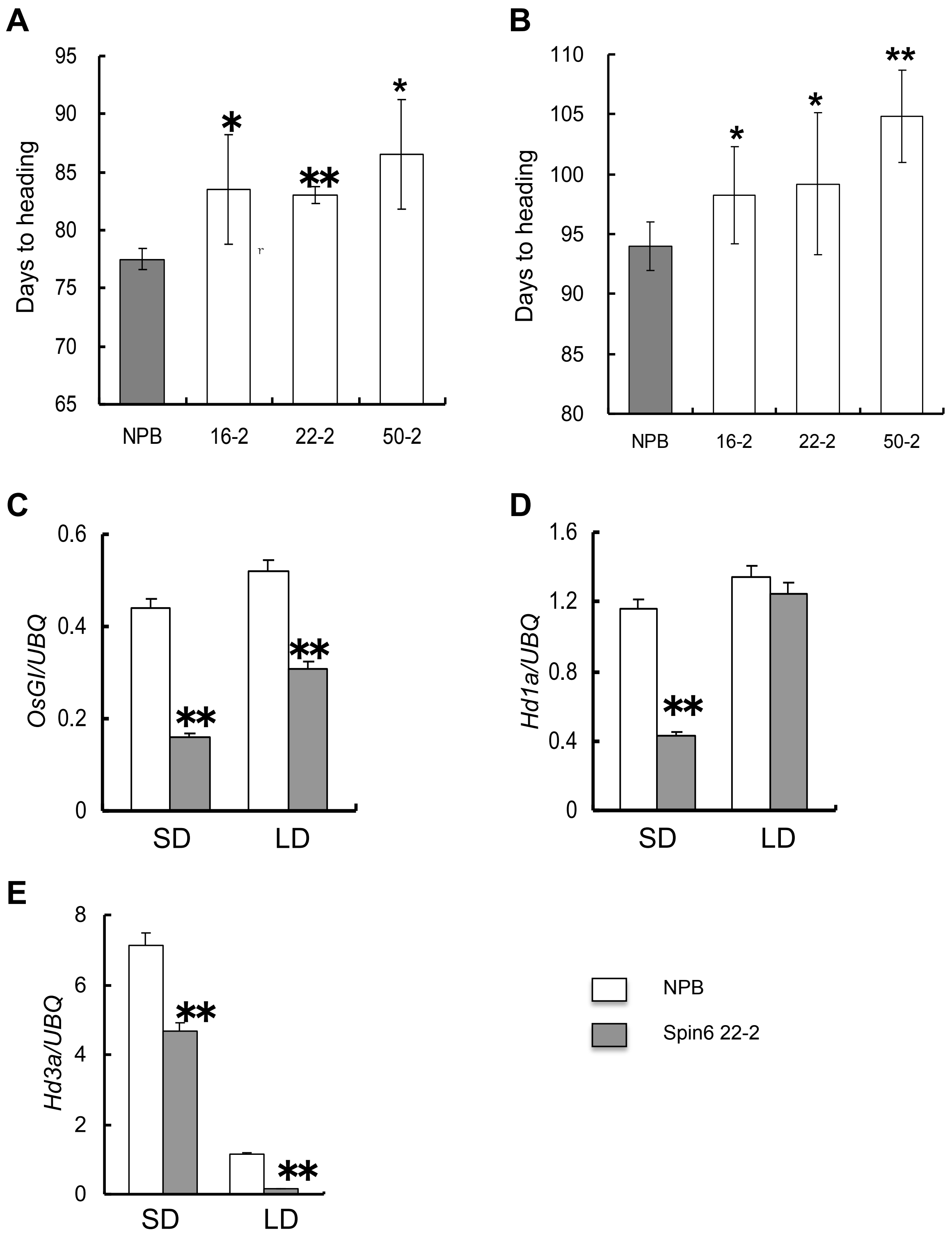

Figure S17. The flowering time of wild type Nipponbare (NPB) and *Spin6* RNAi (22-2) plants under both short day (SD) (A) and long day (LD) (B) conditions, and expression pattern of flowering marker genes *OsGI* (C), *Hd1* (D) and *Hd3a* (E) in both NPB and *Spin6* RNAi plants. The ubiquitin (UBQ) gene was used as the internal control. The data of each lines is the average of 5 plants, error bar represents SD. * and ** represent the significant level at $P<0.05$ and $P<0.001$, respectively.
