# Supplementary material for: The RhoGAP SPIN6 Associates with SPL11 and OsRac1 and Negatively Regulates Programmed Cell Death and Innate Immunity in Rice
Source: PLoS Pathog. 2015 Feb 6;11(2):e1004629. doi: 10.1371/journal.ppat.1004629 (PMC4450066; doi:10.1371/journal.ppat.1004629)
Supplement: S2 Text — Table S1. The cDNA sequence identity of Spin6 and its two rice homologous genes. Table S2. The protein sequence identity of SPIN6 and its two rice homologous genes. Table S3. Primers used in this study. (PDF) [file ppat.1004629.s002.pdf]

Table S1. The cDNA sequence identity of *Spin6* and its two rice homologous genes

	Os07g46450 (Spin6)	Os03g24180 (Spin6.2)	Os03g11140 (Spin6.3)
Os07g46450	100		
Os03g24180	81.7	100	
Os03g11140	61.4	61.7	100

Table S2. The protein sequence identity of SPIN6 and its two rice homologous genes

	Os07g46450	Os03g24180	Os03g11140
Os07g46450	100		
Os03g24180	79.9	100	
Os03g11140	40.7	40.8	100

Table S3. Primers used in this study

Gene	Primer name	Primer sequence	Objects
SPIN6	SPIN6URiF	*CACC*GATATTGATGGCAGCCCTTCTTTC	*SPIN6* UTR RNAi constructs making
	SPIN6URiR	ATTCGATTCTCAGCAGTATG	
	SPIN6FL F	ATGGCTGCGGCGACGGCG	*SPIN6* full length cloning
	SPIN6FL R	CTACCTTGGACTGTTCCAAG	
	SPIN6NT F	CGGAGCGGCAGCAGCAACAGC	*SPIN6* N-terminal cloning
	SPIN6NT R	TGACTCTTCACTTCCGCTATCTTCA	
	SPIN6CT F	CAAATTGAAGATAGCGGAAGTGA	*SPIN6* C-terminal cloning
	SPIN6CT R	ATGACGAAGACTTGTATGG	
	SPIN6PH F	CGGAGCGGCAGCAGCAACAGC	*SPIN6* PH domain cloning
	SPIN6PH R	AATCGGCCTACCAACAACCAATGA	
	SPIN6GAP F	GTTGTTGGTAGGCCGATTCTTCTT	*SPIN6* GAP domain cloning
	SPIN6GAP R	TGACTCTTCACTTCCGCTATCTTCA	
	SPIN6QF	GGAAGAAGCTCTTGCACAGG	*SPIN6* Q RT-PCR
	SPIN6QR	CTCTTCTCTCGCCAGTTTGG	
SPL11	SPL11FLF	ATGAAAAAGTTTCAGGGAGT	*SPL11* full length cloning
	SPL11FLR	TCATACAACCATAGGGTATTG	
	SPL11ARM F	CCAGACACTGAGGAGCAGAG	*SPL11* ARM domain cloning
	SPL11 ARM R	CTCTTGTTGCTGGACTAGGAA	
	SPL11QF	ATTCCTGACGAGTTCCGATG	*SPL11* Q RT-PCR
	SPL11QR	CATCTTCTGTTGCGTGGTTG	
OsRac1	OsRac1FL F	ATGAGCTCGGCGGCGGCGGCGACGA	*OsRac1* full length cloning
	OsRac1FL R	CTACGCGAAACAAGCGCTTCCGC	
	OsRac1 QF	GCCCCATGGATCTACTCAGA	*OsRac1* Q RT-PCR
	OsRac1 QR	AAAGGAGCTCCACTGCAGAA	
Ubiquitin	UBQQF	AACCAGCTGAGGCCCAAGA	*Ubiquitin* Q RT-PCR
	UBQQR	ACGATTGATTTAACCAGTCCATGA	
PBZ1	PBZ1QF	CCCTGCCGAATACGCCTAA	*PBZ1* Q RT-PCR
	PBZ1QR	CTCAAACGCCACGAGAATTTG	
PR1a	PR1aQF	CGTCTTCATCACCTGCAACTACTC	*PR1a* Q RT-PCR

	PR1aQR	CATGCATAAACACGTAGCATAGCA	
PAL	PALQF	CTACAACAACGGGCTGACCT	*PAL* Q RT-PCR
	PALQR	TCTGGACATGGTTGGTGATG	
OsSGT1	OsSGT1 QF	TTCGCATCTGGTGACTCAAG	*OsSGT1* Q RT-PCR
	OsSGT1 QR	TGCTCCATCTTCAGCCTTCT	
OsRAR1	OsRAR1 QF	GTTGAGAAGCCTGCAGTTCC	*OsRAR1* Q RT-PCR
	OsRAR1 QR	CAGGTCCTGGATGGTAATCG	
OsRbohB	OsrbohB QF	GTTCTACTGTGGTGAGCCTGT	*OsRbohB* Q RT-PCR
	OsrbohB QR	CATACACGGGTATTTCTCAA	
OsNAC4	PALQF	TCCTGCCACCATTCTGAGATG	*OsNAC4 Q RT-PCR*
	PALQR	TTGCAGAATCATGCTTGCCAG	
OsPR5	OsPR5 QF	CAACAGCAACTACCAAGTCGTCTT	*OsPR5* Q RT-PCR
	OsPR5 QR	CAAGGTGTCGTTTTATTCATCAAC	
